# Genetic Convergence of Autism Spectrum Disorder and Epilepsy: From Molecular Mechanisms to Precision Therapeutics

**DOI:** 10.3390/genes17101308

**Published:** 2026-10-20

**Authors:** Iliyana Pacheva

**Affiliations:** 1 Department of Pediatrics, Medical University of Plovdiv, 4002 Plovdiv, Bulgaria; iliyana.pacheva@mu-plovdiv.bg; 2 University Hospital “St. George”, 4000 Plovdiv, Bulgaria

**Keywords:** autism spectrum disorder, epilepsy, genetic convergence, neurodevelopmental disorders, excitation–inhibition balance, precision medicine, precision therapeutics, developmental and epileptic encephalopathy

## Abstract

Autism spectrum disorder (ASD) and epilepsy frequently co-occur, with the clinical overlap particularly evident in individuals with intellectual disability, developmental impairment, and genetically defined neurodevelopmental disorders. Advances in genomic diagnostics have revealed substantial genetic and molecular convergence between ASD and epilepsy. This convergence encompasses both recurrent copy-number variants (CNVs), including 15q11–q13, 16p11.2, and 22q11.2 rearrangements, and sequence-level alterations in genes implicated in both epilepsy and ASD-related neurodevelopmental phenotypes, including *SCN1A*, *SCN2A*, *GRIN2A*, *SYNGAP1*, *STXBP1*, *TSC1/TSC2*, *MECP2*, and *UBE3A*. These diverse genetic alterations affect gene dosage, ion-channel function, synaptic organization and neurotransmission, intracellular signaling, and gene regulation, ultimately converging on disruption of circuit homeostasis, excitation–inhibition regulation, synaptic plasticity, and network dynamics. Importantly, the relationship between epilepsy and ASD is neither uniform nor necessarily causal: epileptic activity may aggravate neurodevelopmental dysfunction in some disorders, whereas in others, seizures and autistic features represent partially independent consequences of a shared genetic defect. This heterogeneity has major therapeutic implications. Emerging precision approaches increasingly incorporate variant-specific functional effects and include ion-channel modulation, pathway-directed treatments, metabolic correction, and gene- or RNA-targeted therapies. However, successful seizure control does not necessarily translate into normalization of neurodevelopmental outcomes, emphasizing the importance of developmental timing and of distinguishing seizure-directed benefit from modification of the underlying neurodevelopmental disorder. This review integrates clinical, genetic, molecular, cellular, and network-level evidence linking ASD and epilepsy and examines how these convergent mechanisms may guide precision therapeutics and future early-intervention strategies.

## 1. Introduction

Autism spectrum disorder (ASD) is a heterogeneous neurodevelopmental condition defined according to the Diagnostic and Statistical Manual of Mental Disorders, Fifth Edition, Text Revision (DSM-5-TR), by persistent deficits in social communication and social interaction across multiple contexts, together with restricted, repetitive patterns of behavior, interests, or activities. A formal diagnosis requires the presence of all three social-communication criteria and at least two of the four restricted/repetitive behavior criteria; symptoms must be present in the early developmental period, cause clinically significant impairment, and not be better explained by intellectual disability or global developmental delay [1]. ASD may co-occur with intellectual disability; however, social-communication deficits must exceed those expected for the individual’s general developmental level.

Throughout this review, the term ASD is used when referring to formally diagnosed autism spectrum disorder or to evidence specifically derived from ASD cohorts. The term ‘autistic features’ refers to reported social-communication and/or restricted or repetitive behavioral characteristics resembling components of the ASD phenotype when the available evidence does not establish a formal ASD diagnosis. In genetically defined neurodevelopmental disorders, particularly severe developmental and epileptic encephalopathies (DEEs), such features may occur within a broader neurodevelopmental phenotype and should not be interpreted as synonymous with formally diagnosed ASD. Accordingly, the terms ‘autistic features,’ ‘ASD-related neurodevelopmental dysfunction,’ and ‘broader neurodevelopmental phenotype’ are used throughout the review according to the level and nature of the available phenotypic evidence.

Beyond its defining behavioral features, ASD is frequently accompanied by neurological and neurodevelopmental comorbidities that contribute substantially to clinical heterogeneity and long-term outcome. Among these, epilepsy is particularly important because of its association with intellectual disability (ID), developmental impairment, and increased clinical complexity [2,3].

The relationship between ASD and epilepsy extends beyond simple clinical co-occurrence. Advances in genomic medicine have revealed substantial overlap in their genetic architecture, with pathogenic variation affecting ion channels, synaptic proteins, neurotransmitter receptors, intracellular signaling pathways, and regulators of gene expression and chromatin organization. Many of these molecular defects can simultaneously influence neuronal excitability and the developmental organization of neural circuits, providing a biological framework through which epilepsy and ASD-related neurodevelopmental dysfunction may emerge from a shared molecular substrate [4,5].

Importantly, the relationship between the two phenotypes is neither uniform nor necessarily unidirectional. In some genetic disorders, epileptic activity may itself interfere with cognition and development, whereas in others, seizures and autistic features may represent partially independent consequences of an underlying molecular defect. Developmental timing, variant-specific functional effects, cell-type vulnerability, and compensatory network mechanisms further modify this relationship. Understanding this heterogeneity is increasingly important as genetic diagnosis begins to inform mechanism-based and molecularly targeted therapies [4,6].

This review examines the genetic convergence between ASD and epilepsy across molecular, cellular, and network levels. This review focuses on representative genetic disorders that illustrate major mechanistic pathways, evaluates how these mechanisms converge on altered circuit homeostasis and network function, and discusses their implications for precision therapeutics. Particular attention is given to the variable relationship between seizure control and neurodevelopmental outcomes and to the extent to which emerging precision therapies may modify the underlying disease process rather than seizures alone.

### 1.1. Epidemiological Scale and Phenotypic Modifiers of the Comorbidity

Epilepsy occurs substantially more frequently among autistic individuals than in the general population, although prevalence estimates vary widely according to age, intellectual functioning, ascertainment strategy, and clinical setting. An updated systematic review and meta-analysis reported a pooled epilepsy prevalence of approximately 10% among autistic individuals, with estimates of approximately 7% in cohort studies, 9% in population-based cross-sectional studies, and 19% in clinically ascertained cross-sectional samples [3]. Epilepsy prevalence also increases with age and is higher among individuals with ID and in females, illustrating the importance of phenotypic composition when comparing ASD cohorts [2,3].

Clinical cohorts enriched for neurological comorbidity may consequently show substantially higher rates. In a previous pediatric hospital-based cohort from our group, epilepsy was identified in 44.4% of children with ASD; however, the cohort was characterized by a high prevalence of ID and should therefore not be interpreted as representative of population-level prevalence [7]. Such observations illustrate the marked clinical heterogeneity of the ASD–epilepsy association and the importance of distinguishing population-based estimates from those derived from neurologically selected cohorts. Higher prevalence estimates in clinically ascertained samples likely reflect enrichment for individuals with greater neurodevelopmental and neurological complexity, whereas population-based studies include a broader and less clinically selected spectrum of autistic individuals [3].

Intellectual disability represents one of the most consistently demonstrated modifiers of epilepsy risk in ASD. Earlier meta-analytic evidence showed epilepsy in approximately 21.5% of autistic individuals with ID compared with 8% in those without ID, while more recent analyses continue to identify intellectual functioning as a major source of between-study heterogeneity [2,3]. Female sex and increasing age are also associated with higher epilepsy prevalence, suggesting that the classical male predominance observed in ASD cannot be directly extrapolated to the subgroup with epilepsy [2,3].

Together, these findings argue against a single fixed estimate of epilepsy prevalence in ASD. Instead, the co-occurrence should be regarded as a phenotype strongly modified by intellectual function, age, sex, ascertainment, and underlying etiology.

### 1.2. Clinical Overlap and Distinctive Manifestations of ASD and Epilepsy

Autism spectrum disorder and epilepsy are clinically distinct disorders despite their frequent co-occurrence. As defined above, ASD is characterized by persistent deficits in social communication and social interaction together with restricted, repetitive patterns of behavior, interests, or activities [1]. Epilepsy, by contrast, is diagnosed according to established clinical criteria reflecting an enduring predisposition to generate epileptic seizures [8]. Their coexistence should therefore not be interpreted as a single clinical phenotype.

The clinical intersection between ASD and epilepsy becomes particularly relevant in individuals with broader neurodevelopmental impairment. Intellectual disability is one of the most consistently demonstrated modifiers of epilepsy risk in ASD and contributes substantially to the heterogeneity of the combined phenotype [2,3]. Developmental regression may also occur in a subset of individuals with ASD, most commonly involving loss of previously acquired language and/or social skills [9]. However, developmental abnormalities and regression are heterogeneous phenomena and should not be assumed to reflect the same underlying mechanism across different neurodevelopmental disorders.

This distinction is particularly important in genetically defined neurodevelopmental disorders and developmental and epileptic encephalopathies (DEEs). In many genetic DEEs, the underlying molecular defect may itself contribute directly to developmental impairment, in addition to the potential adverse contribution of epileptic activity [10,11]. Consequently, developmental delay or regression in these disorders should not automatically be attributed to seizures or epileptiform activity alone. Similarly, autistic features or ASD-related behaviors occurring within complex genetic syndromes may show syndrome-specific characteristics and may differ from nonsyndromic ASD; severe intellectual or global developmental impairment may further complicate formal ASD assessment [12].

Core ASD diagnostic criteria concern social-communication and social-interaction deficits and restricted/repetitive patterns of behavior rather than neurological motor manifestations [1]. Motor abnormalities, hypotonia, movement disorders, and other neurological manifestations may instead occur within the broader phenotype of genetically defined neurodevelopmental and epileptic disorders [10]. When such manifestations accompany autistic features and epilepsy, they should therefore be interpreted within the broader neurological and genetic phenotype rather than as core manifestations of ASD [10,12].

Overall, the clinical overlap between ASD and epilepsy is best understood as a heterogeneous neurodevelopmental context in which distinct core phenotypes may coexist with shared developmental modifiers and manifestations of an underlying genetic disorder. The principal shared and distinctive clinical manifestations relevant to this spectrum are summarized in Table 1.

## 2. The Genetic Architecture and Diagnostic Yield in the ASD–Epilepsy Spectrum

Advances in chromosomal microarray analysis (CMA), next-generation sequencing (NGS), whole-exome sequencing (WES), and increasingly whole-genome sequencing (WGS) have substantially increased the ability to identify molecular etiologies in neurodevelopmental disorders. However, reported diagnostic yields vary considerably and should not be interpreted as fixed estimates applicable across all ASD or epilepsy populations. Differences in cohort composition and phenotypic severity, particularly the presence of ID, developmental impairment, syndromic features, and early-onset epilepsy, together with differences in sequencing strategy and variant interpretation, contribute substantially to this variability [13,14,15].

Early studies already demonstrated the influence of phenotypic complexity. In the study by Tammimies et al., combined CMA and WES produced an overall molecular diagnostic yield of 15.8% in children with ASD, with substantially higher yield among children with more complex morphological phenotypes [13]. This observation is consistent with subsequent evidence indicating that genetically enriched or clinically more complex neurodevelopmental cohorts generally have higher diagnostic yields than less selected ASD populations.

This pattern was confirmed in a systematic review and meta-analysis of 103 studies comprising 32,331 individuals, in which clinical sequencing yielded a molecular diagnosis in 17.1% of ASD cohorts, compared with 24.0% of epilepsy cohorts and 28.2% of ID cohorts. Within epilepsy populations, yield was higher in individuals with ID (27.9%) and reached 36.8% in early-onset seizures [14]. Thus, differences between diagnostic-yield estimates partly reflect differences in the underlying clinical populations rather than differences in test performance alone.

More recent evidence also demonstrates the impact of sequencing strategy. A 2025 systematic review and meta-analysis of 416 studies encompassing 124,937 individuals found substantially higher diagnostic yields for NGS-based approaches than for CMA. WES yielded molecular diagnoses in approximately 35.3% of NDD cohorts and 34.2% of epilepsy cohorts, while NDD accompanied by epilepsy had a pooled diagnostic yield of 35.6%. Dysmorphic features, syndromic presentations, early-onset epilepsy, and epileptic encephalopathy were associated with above-average yields [15]. The higher estimates in this broader meta-analysis should therefore not be interpreted as directly contradicting earlier ASD-specific estimates; rather, they reflect differences in cohort composition, phenotype severity, and the diagnostic technologies evaluated.

Taken together, these studies suggest that diagnostic yield across the ASD–epilepsy spectrum is best considered as a context-dependent range rather than a universal value. Lower yields are generally observed in less selected ASD cohorts and with approaches such as CMA, whereas substantially higher yields may be achieved with sequencing-based testing in individuals with complex neurodevelopmental phenotypes, ID, syndromic features, or early-onset epilepsy [13,14,15]. Direct comparison between studies nevertheless remains limited by substantial methodological and clinical heterogeneity, including differences in patient ascertainment and phenotypic composition, sequencing strategy and technical platform, genes included in targeted panels, analytical approaches such as trio-based versus proband-only sequencing and variant filtering, and the criteria used for variant classification and interpretation [14,15].

### 2.1. Epilepsy as a Marker of Increased Genetic Complexity in ASD

Within ASD populations, epilepsy should therefore be regarded not as proof of a monogenic disorder but as an important clinical feature that may increase suspicion of an identifiable genetic etiology, particularly when accompanied by ID, developmental regression, dysmorphism, abnormal head growth, early seizure onset, or other neurological abnormalities [14,16].

This association is supported by recent ASD-specific data. In a cohort of 523 children with ASD, an explanatory genetic diagnosis was obtained in 15.1% overall, with WES outperforming CMA (23.0% vs. 8.3%). Epilepsy was significantly more frequent among children with genetically defined ASD than among those without an identified genetic diagnosis (35.4% vs. 16.4%), and seizure onset occurred at a younger age in the genetically defined group. These findings support epilepsy as a clinically relevant marker of genetic enrichment while also demonstrating that it is neither necessary nor sufficient for establishing a molecular diagnosis [16].

### 2.2. Structural and Sequence-Level Genetic Variation

The shared genetic architecture of ASD and epilepsy encompasses both structural genomic variation and sequence-level pathogenic variants. CMA remains particularly effective for detecting pathogenic copy-number variants (CNVs), whereas WES and WGS substantially expand detection of single-nucleotide variants and small insertions or deletions. These approaches are therefore complementary rather than conceptually interchangeable [13].

Recurrent CNVs associated with neurodevelopmental phenotypes—including abnormalities involving 15q11–q13, 16p11.2, and 22q11.2—illustrate the complexity of genotype–phenotype relationships. Their manifestations are variably penetrant and may include ASD, ID, language impairment, epilepsy, and other neurological or systemic abnormalities. Importantly, neither epilepsy nor ASD is obligatory across all carriers, and phenotype severity may differ according to genomic architecture, parental origin, dosage, and additional genetic modifiers. This variability argues against describing individual CNVs as uniformly producing a fixed ASD–epilepsy phenotype [17,18,19].

Recurrent copy-number variants (CNVs) represent an important component of the genetic architecture shared by ASD, epilepsy, and related neurodevelopmental disorders. Several recurrent genomic regions, including 1q21.1, 15q11–q13, 15q13.3, 16p11.2, 16p13.11, 22q11.2, and 22q13.3, have been associated with variable combinations of ASD or autistic features, epilepsy, intellectual/developmental impairment, and other neurodevelopmental manifestations [17,18,19,20,21,22,23]. Importantly, the phenotypic consequences of reciprocal deletions and duplications are not necessarily equivalent. This is particularly evident for 16p11.2 and 22q11.2, where reciprocal dosage changes are associated with differences in penetrance and in the relative expression of neurodevelopmental and neurological manifestations [18,19,24,25,26]. In addition, recurrent CNVs frequently show incomplete penetrance and variable expressivity, indicating that genomic dosage alone does not fully determine the individual phenotype [18,20,26]. Common recurrent CNVs relevant to the ASD–epilepsy spectrum and their principal clinical associations are summarized in Table 2.

Collectively, these CNVs illustrate several features of the genetic convergence between ASD and epilepsy. First, the same genomic imbalance may be associated with a broad spectrum of neurodevelopmental outcomes rather than a single disorder. Second, reciprocal dosage changes within the same locus may produce partially distinct phenotypic profiles, as exemplified by 16p11.2 and 22q11.2 deletions and duplications [18,19,24,25,26]. Third, many recurrent CNVs encompass multiple dosage-sensitive genes, making attribution of the complete phenotype to a single gene inappropriate. Nevertheless, individual genes within these regions, including *UBE3A* within 15q11–q13, *CHRNA7* and *OTUD7A* within 15q13.3, and *SHANK3* within 22q13.3, provide important mechanistic links to pathways involved in synaptic function, neuronal excitability, and neurodevelopment [17,21,23].

Sequence-level pathogenic variants likewise span multiple functional classes. Large-scale genomic studies of neurodevelopmental disorders demonstrate enrichment of damaging variants in genes involved in neuronal excitability, synaptic function, transcriptional regulation, and chromatin remodeling. Within the ASD–epilepsy spectrum, representative examples include voltage-gated ion-channel genes (*SCN1A*, *SCN2A*, *SCN8A*, *KCNQ2*, and *KCNT1*), synaptic genes (*SHANK3*, *SYNGAP1*, and *STXBP1*), neurotransmitter-receptor genes (*GRIN2A*, *GRIN2B*, and *GABRB3*), and regulators of intracellular signaling and gene expression (*TSC1/TSC2*, *PTEN*, *MECP2*, *UBE3A*, *FMR1*, and *CHD8*) [4,32,33].

The clinical implication is therefore not that epilepsy in ASD automatically indicates a “druggable genetic syndrome,” but that co-occurring epilepsy—particularly when early-onset or accompanied by ID, developmental regression, dysmorphism, or other neurological features—strengthens the rationale for comprehensive genomic evaluation. Molecular diagnosis can then provide the basis for mechanism-informed interpretation and, in selected disorders, precision therapeutic strategies [14,15,16].

## 3. Functional Classification and Molecular Mechanisms of Shared Genes

Despite their considerable functional diversity, genes associated with ASD and epilepsy converge on a limited set of neurodevelopmental processes. These include regulation of intrinsic neuronal excitability and excitation–inhibition balance, synaptic formation and transmission, activity-dependent signaling and synaptic plasticity, and the maturation and homeostatic regulation of neural circuits [5,34,35]. Importantly, ASD-related circuit dysfunction should not be interpreted simply as a uniform shift toward excessive excitation. Genetic perturbations may alter excitatory or inhibitory neurons, synaptic strength, intrinsic excitability, or compensatory homeostatic mechanisms in a cell type-, circuit-, and developmental stage-dependent manner [34,35]. Neuronal activity itself is a critical regulator of synapse development, stabilization, elimination, and activity-dependent gene expression; consequently, genetic disturbances of neuronal excitability or synaptic signaling during sensitive developmental periods may alter circuit maturation and plasticity, providing a mechanistic route to ASD-related neurodevelopmental dysfunction [5,36]. The same molecular and cellular processes regulate network stability and synchronization and, when sufficiently disrupted, may also promote epileptogenesis. Thus, ASD and epilepsy may represent parallel and interacting consequences of disturbed circuit development and homeostasis rather than a simple unidirectional relationship in which one disorder necessarily causes the other. Representative genes illustrating these convergent molecular and cellular mechanisms are summarized in Table 3.

### 3.1. Voltage-Gated Ion Channelopathies

Voltage-gated sodium and potassium channels are major determinants of intrinsic neuronal excitability, action-potential generation, and repetitive firing. Pathogenic variants in genes encoding these channels provide a particularly direct link between altered neuronal excitability and the overlapping epilepsy–neurodevelopmental spectrum. Importantly, channel dysfunction may influence not only seizure susceptibility but also activity-dependent circuit maturation and plasticity, thereby providing a mechanistic route to ASD-related neurodevelopmental dysfunction. The resulting phenotype cannot be predicted simply from whether channel activity is increased or decreased, but depends on the direction and magnitude of the functional alteration, the affected neuronal population and subcellular compartment, and the developmental stage at which channel dysfunction alters circuit function [39,41].

- *SCN1A* encodes the voltage-gated sodium-channel α-subunit NaV1.1, which is particularly important for the excitability of GABAergic inhibitory interneurons. In Dravet syndrome, most pathogenic *SCN1A* variants result in loss of function. Experimental models have demonstrated impaired sodium currents and reduced excitability of inhibitory interneurons, providing a mechanistic basis for network disinhibition and seizure susceptibility [37]. Thus, loss of sodium-channel function at the cellular level may paradoxically produce network hyperexcitability when the predominantly affected neuronal population is inhibitory. Importantly, the same interneuron dysfunction may contribute directly to the neurodevelopmental phenotype rather than acting exclusively through recurrent seizures. In Scn1a-haploinsufficient mice, impaired GABAergic neurotransmission is associated with deficits in social behavior and cognition, while selective reduction in NaV1.1 in forebrain interneurons is sufficient to reproduce these abnormalities. Their pharmacological rescue by enhancement of GABAergic transmission further supports disrupted inhibitory circuit function as a mechanistic link between *SCN1A* dysfunction and ASD-related behavioral phenotypes [38].
- *SCN2A*, encoding NaV1.2, illustrates a particularly clear relationship between variant function, developmental timing, and divergent neurological phenotypes. Gain-of-function variants are most strongly associated with neonatal or early-infantile epileptic phenotypes, in which enhanced NaV1.2 activity increases the excitability of developing excitatory neurons, whereas many loss-of-function variants are associated with ASD, intellectual disability, and later-onset neurodevelopmental phenotypes, sometimes without epilepsy [39,74]. Importantly, the role of NaV1.2 extends beyond action-potential generation. In cortical pyramidal neurons, NaV1.2 contributes to dendritic excitability and action-potential backpropagation, thereby influencing synaptic strength and activity-dependent plasticity. Experimental reduction in NaV1.2 function impairs dendritic backpropagation and synaptic plasticity, providing a mechanistic link between *SCN2A* haploinsufficiency and ASD-related neurodevelopmental dysfunction [40]. Thus, *SCN2A*-related epilepsy and ASD may arise from distinct but developmentally related consequences of altered NaV1.2 function, with the phenotype shaped by the direction and magnitude of channel dysfunction, neuronal compartment, and developmental stage.
- *SCN8A* encodes NaV1.6, a voltage-gated sodium channel highly enriched at the axon initial segment and nodes of Ranvier and critically involved in action-potential initiation, repetitive firing, and propagation. Many pathogenic missense variants associated with early developmental and epileptic encephalopathy produce gain-of-function effects, including enhanced persistent or resurgent sodium currents, thereby promoting neuronal hyperexcitability and seizure susceptibility [41,75]. However, the functional and phenotypic spectrum of *SCN8A*-related disorders extends beyond gain-of-function epileptic encephalopathy, and loss-of-function or functionally complex variants may be associated with intellectual disability, autistic features, and other neurodevelopmental phenotypes with variable seizure involvement [42,76]. Experimental evidence further supports convergence at the level of neuronal and circuit excitability: an Scn8a mouse model carrying the R1620L variant exhibits altered neuronal excitability and spontaneous seizures together with impaired social behavior and learning, linking NaV1.6 dysfunction to both epileptic and ASD-related phenotypes [77]. Thus, *SCN8A*-related epilepsy and neurodevelopmental dysfunction may represent partially overlapping consequences of disturbed NaV1.6-dependent neuronal and network function rather than a simple sequence in which seizures alone produce the developmental phenotype.

Potassium-channel disorders further illustrate how both reduced and enhanced channel activity can disrupt developing neural circuits.

- *KCNQ2* encodes the KV7.2 subunit, which contributes to the neuronal M-current that stabilizes membrane potential and limits repetitive firing. Loss-of-function variants reduce M-current and are associated with a spectrum ranging from self-limited neonatal epilepsy to severe developmental and epileptic encephalopathy, with dominant-negative effects particularly associated with severe phenotypes [43]. Importantly, *KCNQ2* dysfunction may affect neurodevelopment through mechanisms that extend beyond seizure generation. Human iPSC-derived neuronal models demonstrate that reduced M-current can alter membrane potential, action-potential generation, homeostatic conductance, and early network oscillations, resulting in delayed neuronal maturation and disrupted network development [44,45]. Conversely, specific gain-of-function variants have been associated with developmental disability, prominent language impairment, and autistic features, sometimes in the absence of neonatal seizures [46]. Thus, both insufficient and excessive KV7.2 activity may disturb the developmental regulation of neuronal excitability and circuit maturation, providing a mechanistic link between *KCNQ2* dysfunction, epilepsy, and ASD-related neurodevelopmental phenotypes.
- *KCNT1* encodes the sodium-activated potassium channel KNa1.1 (Slack), which contributes to the regulation of firing adaptation and neuronal excitability during repetitive activity. Pathogenic variants associated with severe early-onset epilepsies, particularly epilepsy of infancy with migrating focal seizures, commonly produce gain-of-function effects [47]. Although increased potassium conductance might be expected to suppress neuronal firing, *KCNT1* gain of function can paradoxically promote network hyperexcitability by altering action-potential repolarization, firing patterns, and neuronal subtype-specific excitability. The neurodevelopmental consequences of *KCNT1* dysfunction may also extend beyond the effects of recurrent seizures. Slack channels participate in signaling pathways relevant to neuronal plasticity and interact with the fragile X messenger ribonucleoprotein FMRP, suggesting a potential link between *KCNT1*-dependent excitability, activity-dependent signaling, and cognitive development [48]. Experimental manipulation of Kcnt1 further produces abnormalities in learning-related behavior and seizure susceptibility, supporting a broader role for Slack channels in both network excitability and neurodevelopment [49]. However, a direct *KCNT1*-specific mechanism for ASD remains insufficiently established, and the neurodevelopmental phenotype is therefore better interpreted as reflecting disruption of excitability and neuronal plasticity rather than a defined ASD-specific pathway.

Collectively, sodium- and potassium-channel disorders demonstrate that the shared pathophysiological substrate of epilepsy and neurodevelopmental dysfunction cannot be reduced to a simple increase or decrease in neuronal excitation. Variant-specific alterations in ion-channel function may disrupt intrinsic excitability, inhibitory control, dendritic integration, activity-dependent plasticity, and the maturation and temporal organization of developing neural circuits. Depending on the affected channel, neuronal population, subcellular compartment, and developmental stage, these disturbances may promote epileptic hypersynchrony, ASD-related neurodevelopmental dysfunction, or both. Ion channelopathies therefore provide a particularly clear example of how a primary molecular defect can generate partially overlapping epileptic and neurodevelopmental phenotypes through disruption of circuit homeostasis.

### 3.2. Synaptic Scaffolding, Presynaptic Release, and Activity-Dependent Synaptic Regulation

A second major group of genes shared between ASD and epilepsy affects synaptic organization, maturation, plasticity, and neurotransmitter release. Unlike ion channelopathies, in which altered intrinsic membrane excitability provides the primary molecular disturbance, synaptic disorders can disrupt the formation, strength, timing, and experience-dependent refinement of neuronal connections. These abnormalities may interfere directly with developmental circuit assembly and cognitive or social function, while concomitant disturbances in excitatory–inhibitory regulation and network stability may increase seizure susceptibility. Synaptic dysfunction therefore provides another mechanistic route through which a single genetic defect can generate partially overlapping ASD-related neurodevelopmental and epileptic phenotypes [5].

- *SHANK3* encodes a multidomain scaffolding protein of the postsynaptic density at excitatory glutamatergic synapses. *SHANK3* haploinsufficiency, classically associated with Phelan–McDermid syndrome, disrupts postsynaptic organization and alters glutamatergic synaptic transmission, dendritic-spine structure, and activity-dependent plasticity. In Shank3-deficient mice, reduced AMPA receptor-mediated transmission and impaired long-term potentiation are accompanied by abnormalities in social interaction and communication, directly linking postsynaptic dysfunction to ASD-related behavioral phenotypes [31,55,78]. Studies of patient-derived neurons similarly demonstrate impaired excitatory synaptic transmission and altered glutamate-receptor function, supporting a primary synaptic mechanism for the neurodevelopmental phenotype. Epilepsy occurs only in a subset of individuals with *SHANK3*-related disorders [6], indicating that profound disruption of excitatory synaptic development is sufficient to produce neurodevelopmental dysfunction but does not invariably cross the threshold for epileptic network instability. Thus, in *SHANK3* alterations, ASD-related dysfunction and epilepsy are better regarded as variably expressed consequences of abnormal synaptic and circuit organization rather than as obligatorily coupled phenotypes.
- *SYNGAP1* encodes SynGAP, a postsynaptic Ras/Rap GTPase-activating protein highly enriched at excitatory glutamatergic synapses, where it regulates signaling downstream of NMDA receptors and controls synaptic maturation and plasticity. *SYNGAP1* haploinsufficiency is associated with intellectual disability, epilepsy, and frequent ASD-related behavioral features. Experimental studies have shown that reduced SynGAP expression accelerates dendritic-spine maturation and the functional development of excitatory synapses during critical postnatal periods, resulting in premature stabilization of neural circuits and impaired experience-dependent synaptic plasticity [56,57]. These developmental abnormalities provide a direct mechanistic link to persistent cognitive and behavioral dysfunction. At the same time, altered excitatory synaptic function and disruption of excitation–inhibition regulation increase network excitability and seizure susceptibility [56,57,58,79]. Thus, *SYNGAP1* haploinsufficiency provides a particularly clear example of mechanistic convergence between ASD-related neurodevelopmental dysfunction and epilepsy: both phenotypes arise from disruption of the timing and plasticity of excitatory circuit development, rather than one necessarily being secondary to the other.

In contrast to these predominantly postsynaptic mechanisms, *STXBP1* encodes syntaxin-binding protein 1 (Munc18-1), an essential component of the presynaptic machinery controlling synaptic vesicle fusion and neurotransmitter release. *STXBP1* haploinsufficiency disrupts synaptic transmission and causes a broad neurodevelopmental disorder characterized by developmental delay or intellectual disability, epilepsy, movement disorders, and variable behavioral abnormalities. Because Munc18-1 is required at both excitatory and inhibitory synapses, the consequences of *STXBP1*-related dysfunctions cannot be attributed to a selective defect of a single neurotransmitter system. Nevertheless, experimental models demonstrate particular vulnerability of inhibitory circuitry: Stxbp1 haploinsufficiency reduces cortical inhibitory neurotransmission through distinct abnormalities affecting parvalbumin- and somatostatin-expressing interneurons, resulting in cortical hyperexcitability and increased seizure susceptibility [59]. The same models exhibit cognitive and behavioral abnormalities, including impaired learning and repetitive behaviors, supporting a broader effect of presynaptic dysfunction on neurodevelopmental circuit function. Thus, *STXBP1* alterations provide a mechanistic link between impaired neurotransmitter release, disturbed excitation–inhibition regulation, and the coexistence of epileptic and neurodevelopmental phenotypes, although linking these alterations to ASD-related features remain incompletely understood.

- *CDKL5* encodes a brain-enriched serine/threonine kinase that regulates phosphorylation-dependent processes involved in neuronal maturation, dendritic morphogenesis, synapse formation, and synaptic function. Pathogenic loss-of-function variants cause *CDKL5* deficiency disorder (CDD), a severe developmental and epileptic encephalopathy characterized by very early-onset, frequently drug-resistant seizures and profound neurodevelopmental impairment. Autistic features are reported in CDD, although formal ASD diagnosis can be difficult in individuals with severe global developmental impairment. Experimental data indicate that loss of CDKL5 function disrupts dendritic and synaptic development, supporting a primary neurodevelopmental mechanism that is not reducible to seizure burden alone [60,61]. CDD therefore extends the synaptic convergence model beyond structural scaffolds and vesicle-release proteins to activity-dependent kinase signaling.

Together, *SHANK3*, *SYNGAP1*, *STXBP1*, and *CDKL5* illustrate how disturbances at different levels of synaptic function can converge on a common set of downstream processes linking epilepsy and neurodevelopmental dysfunction. Altered postsynaptic organization and receptor signaling, abnormal timing of excitatory-synapse maturation, defective presynaptic neurotransmitter release, and disrupted activity-dependent kinase signaling can all impair synaptic plasticity, excitation–inhibition regulation, and the developmental refinement of neural circuits [55,56,57,58,59,60,61]. These disturbances may simultaneously interfere with the circuit adaptations required for cognitive, behavioral, and social development and increase susceptibility to pathological network synchronization and seizures. Importantly, however, the strength and nature of this mechanistic convergence differ among genes. *SYNGAP1* provides a particularly clear example in which disruption of excitatory-synapse maturation and critical-period plasticity is linked to both neurodevelopmental dysfunction and seizure susceptibility [56,57,58], whereas in *SHANK3*- and *STXBP1*-related disorders the evidence for a single shared mechanism specifically linking ASD and epilepsy is less direct [55,59]. CDD further illustrates how a primary disturbance of neuronal and synaptic maturation can coexist with severe early-onset epilepsy without implying that the neurodevelopmental phenotype is secondary to seizure activity [60,61]. Thus, synaptic dysfunction represents a convergent biological substrate for ASD-related or broader neurodevelopmental dysfunction and epilepsy, rather than a uniform causal pathway producing both phenotypes in every genetic disorder.

### 3.3. Glutamatergic and GABAergic Receptor Dysfunction

Excitatory glutamatergic and inhibitory GABAergic neurotransmission represent another major point of mechanistic convergence between ASD and epilepsy. Pathogenic variants affecting ligand-gated neurotransmitter receptors can alter receptor expression and trafficking, agonist sensitivity, channel gating, synaptic current kinetics, and activity-dependent plasticity. Because these receptors also regulate synapse maturation and circuit refinement, their dysfunction may directly affect neurodevelopment while simultaneously destabilizing network activity and increasing seizure susceptibility. Importantly, the resulting phenotype cannot be reduced to a simple model of excessive excitation or insufficient inhibition, because both gain- and loss-of-function effects may disrupt circuit function in a receptor-, cell type-, and developmental stage-dependent manner [50,80].

- *GRIN2A* encodes the GluN2A subunit of N-methyl-D-aspartate receptors (NMDARs), which are central to excitatory neurotransmission, synaptic plasticity, learning, and activity-dependent circuit maturation. Pathogenic *GRIN2A* variants are particularly associated with epilepsy–aphasia spectrum disorders and related developmental and epileptic phenotypes, but functional effects are heterogeneous and include both gain- and loss-of-function abnormalities [51,52]. Because GluN2A-containing NMDARs contribute to the developmental maturation of excitatory synapses and experience-dependent plasticity, altered receptor function may impair language- and cognition-related circuit development while also modifying neuronal excitability and seizure susceptibility. Thus, *GRIN2A*-related epilepsy and neurodevelopmental dysfunction can reflect parallel consequences of abnormal NMDAR-dependent synaptic signaling rather than a purely sequential effect of seizures on development.
- *GRIN2B* encodes the GluN2B NMDAR subunit, which is highly expressed during early brain development and plays an important role in neuronal differentiation, dendritic morphogenesis, synaptogenesis, and synaptic plasticity. *GRIN2B*-related disorders commonly present with developmental delay or intellectual disability, behavioral abnormalities including ASD, and variable epilepsy. Pathogenic variants show marked functional heterogeneity, including both gain- and loss-of-function effects that may alter receptor kinetics, agonist sensitivity, surface expression, or downstream signaling [50,53]. Experimental studies further indicate that disease-associated GluN2B variants can interfere directly with neuronal differentiation, dendritic development, synapse formation, and plasticity, providing a mechanistic basis for ASD-related neurodevelopmental dysfunction that is not dependent on seizure activity. The same alterations in NMDAR-mediated synaptic signaling can also disturb network excitability and stability, creating a shared mechanistic substrate for epilepsy and neurodevelopmental impairment [50,53].
- On the inhibitory side, *GABRB3* encodes the β3 subunit of the GABA\(_A\) receptor, a major mediator of fast inhibitory neurotransmission in the developing and mature brain. Pathogenic *GABRB3* variants are associated with a broad phenotypic spectrum ranging from relatively mild epilepsies to severe developmental and epileptic encephalopathies, often accompanied by intellectual disability and behavioral or autistic features. Functional consequences are heterogeneous and include altered receptor expression, trafficking, GABA sensitivity, gating, and current amplitude [54]. Reduced GABAergic signaling can lower seizure threshold through impaired inhibitory control; however, pathogenic *GABRB3* variants are functionally heterogeneous, and developmental and epileptic phenotypes cannot be explained by reduced inhibition alone [54,81]. Thus, disruption of β3-containing GABA\(_A\) receptors may affect both network inhibition and developmental circuit organization, providing a plausible mechanistic link between *GABRB3* dysfunction, epilepsy, and ASD-related neurodevelopmental abnormalities [35,54].

Together, *GRIN2A*, *GRIN2B*, and *GABRB3* illustrate how receptor dysfunction can couple altered synaptic transmission to abnormal circuit development. In glutamatergic disorders, impaired NMDAR-dependent signaling may disrupt synaptic maturation, plasticity, and cognitive circuit formation while also destabilizing excitatory network activity. In GABAergic disorders, altered inhibitory receptor function can affect both seizure threshold and the developmental organization of inhibitory control. These disorders therefore reinforce the concept that excitation–inhibition dysregulation is not a uniform directional shift, but a disturbance in the magnitude, timing, localization, and developmental integration of excitatory and inhibitory signaling that may produce epilepsy, ASD-related neurodevelopmental dysfunction, or both.

The relative contribution of these receptor-level mechanisms to epilepsy and ASD-related neurodevelopmental dysfunction nevertheless differs among genes. *GRIN2A*-related disorders are weighted predominantly toward epilepsy–aphasia and speech/language phenotypes, indicating that disruption of GluN2A-dependent signaling does not uniformly produce an ASD–epilepsy phenotype [51,52]. In contrast, *GRIN2B* dysfunction more consistently affects early neurodevelopment, with developmental delay or intellectual disability and behavioral abnormalities including ASD, whereas epilepsy is variably expressed [50,53]. *GABRB3* further illustrates the functional complexity of receptor-mediated convergence: pathogenic variants can produce heterogeneous effects on receptor expression, trafficking, gating, and GABA sensitivity, demonstrating that developmental and epileptic phenotypes cannot be explained simply by a uniform reduction in inhibition [54]. These genotype-specific differences reinforce the concept that a shared molecular pathway may converge on common processes—synaptic plasticity, developmental excitation–inhibition regulation, and circuit maturation—while producing different relative burdens of epilepsy and ASD-related or broader neurodevelopmental dysfunction.

### 3.4. PI3K–AKT–mTOR Pathway Dysregulation

The PI3K–AKT–mTOR signaling pathway represents a major point of mechanistic convergence between ASD and epilepsy. It regulates protein synthesis, cellular growth, neuronal morphology and migration, synapse formation, and activity-dependent plasticity, thereby influencing both the structural organization and functional maturation of developing neural circuits. Dysregulated signaling can consequently produce abnormal neuronal growth, connectivity, and synaptic plasticity relevant to ASD-related neurodevelopmental dysfunction, while alterations in intrinsic excitability, synaptic transmission, and network organization may simultaneously promote epileptogenesis [62,63].

- TSC1 and *TSC2* encode hamartin and tuberin, respectively, which form a functional complex that negatively regulates mTORC1 signaling. Loss-of-function variants disrupt this inhibitory control, resulting in constitutive mTORC1 activation. Tuberous sclerosis complex (TSC) is consequently associated with a broad neurological phenotype that includes epilepsy, intellectual disability, and neuropsychiatric manifestations, with ASD occurring in a substantial subgroup of affected individuals. Importantly, mTOR hyperactivation can directly alter neuronal growth, morphology, synaptic development, and plasticity, providing a primary molecular mechanism for neurodevelopmental dysfunction that does not require epilepsy as an intermediary [62,63]. At the same time, abnormal neuronal morphology and connectivity, altered synaptic function, and changes in network excitability create a substrate for epileptogenesis, while cortical tubers and surrounding dysplastic tissue provide an additional structural contribution to seizure generation [64].

The relationship between epilepsy and neurodevelopmental dysfunction in TSC is therefore not simply causal or unidirectional. The primary *TSC1/TSC2*–mTOR defect can independently disrupt developmental and synaptic processes relevant to cognition and ASD, whereas early and severe epileptic activity may further modify the developing network and may contribute to less favorable developmental outcomes. Experimental and clinical observations consequently support an interactive model in which primary pathway dysregulation, structural brain abnormalities, and epileptic activity contribute to neurodevelopmental outcome to varying degrees across individuals and developmental stages [62,64].

- *PTEN* acts upstream in the PI3K–AKT–mTOR pathway as a major negative regulator of intracellular signaling. Germline loss-of-function variants are associated with *PTEN* hamartoma tumor syndrome and a neurodevelopmental phenotype frequently characterized by macrocephaly, developmental abnormalities, and ASD, while epilepsy occurs in a subset of affected individuals. Loss of PTEN activity enhances PI3K–AKT–mTOR signaling and disrupts neurogenesis, neuronal growth and migration, dendritic and axonal morphology, synapse formation, connectivity, and synaptic plasticity, providing a direct mechanistic route from impaired PTEN function to ASD-related neurodevelopmental dysfunction [65,66]. Experimental Pten loss also produces neuronal hypertrophy, increased excitatory synaptic input, hyperexcitability, and seizures, demonstrating how the same upstream signaling defect can simultaneously generate structural and functional substrates for epileptogenesis [67].

*TSC1/TSC2*- and *PTEN*-related disorders therefore illustrate how dysregulation of a shared intracellular signaling pathway can simultaneously affect neuronal growth, migration, synaptic organization, plasticity, circuit connectivity, and network excitability. These effects provide partially overlapping but not necessarily identical mechanistic routes to ASD-related neurodevelopmental dysfunction and epilepsy. The relative contribution of primary developmental abnormalities, structural pathology, and epileptic activity differs among disorders and individuals, emphasizing that “mTORopathies” should not be regarded as a uniform biological or clinical entity. Rather, they exemplify how pathway-level convergence can generate divergent phenotypes according to the spatial distribution, magnitude, and developmental timing of signaling dysregulation.

### 3.5. Chromatin, Transcriptional, and Translational Regulation

A further level of convergence occurs upstream of ion channels and synapses, through genes that regulate chromatin organization, transcription, protein turnover, and activity-dependent translation. Unlike variants that directly alter membrane conductance or synaptic receptors, these disorders can modify broad developmental gene-expression programs and thereby affect multiple downstream processes simultaneously. Dysregulation of neuronal differentiation, synapse formation and maturation, activity-dependent plasticity, and excitation–inhibition regulation can consequently generate ASD-related neurodevelopmental dysfunction while also altering circuit stability and seizure susceptibility [5,36].

- *CHD8* encodes an ATP-dependent chromatin-remodeling protein and is one of the most consistently implicated high-confidence ASD-associated genes. Disruptive variants cause a recognizable neurodevelopmental syndrome frequently characterized by ASD, developmental delay or intellectual disability, macrocephaly, and gastrointestinal and sleep abnormalities [73]. *CHD8* regulates broad transcriptional programs involved in neuronal differentiation, axon guidance, synapse development, connectivity, and plasticity, providing a direct mechanistic route from chromatin dysregulation to ASD-related neurodevelopmental dysfunction. Seizures occur in a minority of affected individuals and are not a defining feature of *CHD8*-related disorder. *CHD8* is therefore most informative for the ASD–epilepsy interface at the level of pathway convergence: disruption of an upstream chromatin regulator can alter downstream neurodevelopmental and synaptic programs that overlap with those affected by established epilepsy-associated genes, without necessarily producing epilepsy in every disorder or individual [73].
- *MECP2* encodes methyl-CpG-binding protein 2, a chromatin-associated regulator that links neuronal activity to gene-expression programs required for neuronal maturation and circuit function. Pathogenic loss-of-function variants cause Rett syndrome, characterized by severe neurodevelopmental impairment, loss of acquired skills, communication deficits, stereotypic hand movements, motor abnormalities, and frequent epilepsy. MeCP2 dysfunction disrupts activity-dependent transcription, neuronal and synaptic maturation, connectivity, and experience-dependent plasticity and can alter excitation–inhibition regulation at the circuit level [68]. These effects provide a direct developmental mechanism for cognitive and behavioral dysfunction, while abnormalities of excitatory and inhibitory circuit function can simultaneously promote network instability and seizures. *MECP2*-related disease therefore illustrates how disruption of activity-dependent transcriptional regulation can generate both neurodevelopmental and epileptic phenotypes through partially overlapping effects on circuit maturation and homeostasis.
- *UBE3A* encodes an E3 ubiquitin ligase with a parent-of-origin-dependent expression pattern in neurons, where the paternal allele is largely silenced. Loss of function of the maternally expressed neuronal *UBE3A* allele causes Angelman syndrome, characterized by severe developmental impairment, minimal or absent speech, characteristic behavioral features, motor dysfunction, and a high prevalence of epilepsy. UBE3A deficiency disrupts protein homeostasis, synaptic development and plasticity, and neuronal excitability, providing a mechanistic basis for both developmental dysfunction and network instability. Conversely, maternally derived duplications of chromosome 15q11–q13 are strongly associated with ASD, developmental impairment, and epilepsy. However, the duplicated interval contains multiple dosage-sensitive genes, including GABAA-receptor subunit genes; consequently, the Dup15q phenotype cannot be attributed exclusively to increased UBE3A dosage. Disorders involving altered *UBE3A* gene expression or copy number therefore illustrate how genomic imprinting and dosage imbalance can influence synaptic and circuit development while producing markedly different neurodevelopmental and epileptic phenotypes [69,70].
- Finally, *FMR1* encodes fragile X messenger ribonucleoprotein (FMRP), an RNA-binding protein that regulates the translation of numerous neuronal and synaptic mRNAs in response to activity. Loss of FMRP in fragile X syndrome produces dysregulated synaptic protein synthesis and alters synapse maturation and activity-dependent plasticity. These abnormalities provide a direct molecular link to intellectual disability and ASD-related behavioral phenotypes by disrupting the experience-dependent modification of developing neural circuits. Altered synaptic signaling and circuit excitability may also contribute to the increased susceptibility to seizures observed in a subset of affected individuals. FMRP-related translational dysregulation therefore illustrates how abnormal control of activity-dependent protein synthesis can simultaneously influence neurodevelopmental plasticity and network stability [71,72].

Taken together, *CHD8*, *MECP2*, *UBE3A*, and *FMR1* demonstrate that ASD–epilepsy convergence may originate upstream of membrane excitability and synaptic transmission. Disturbances in chromatin remodeling, transcriptional regulation, protein homeostasis, and activity-dependent translation can alter neuronal differentiation, synaptic maturation, receptor and channel expression, connectivity, and circuit plasticity. These upstream abnormalities may therefore converge on many of the same cellular and network processes that are affected more directly by ion-channel, synaptic, and receptor disorders, while the relative expression of ASD-related neurodevelopmental and epileptic phenotypes remains gene- and context-dependent.

Across these mechanistic categories, no single molecular abnormality explains the coexistence of ASD and epilepsy. Pathogenic variants may primarily alter intrinsic membrane excitability, synaptic architecture and neurotransmitter release, receptor-mediated signaling, intracellular growth pathways, or transcriptional and translational programs. Yet these apparently distinct molecular defects converge on a smaller set of systems-level consequences: impaired circuit homeostasis, disturbed excitation–inhibition regulation, altered synaptic plasticity, and abnormal maturation and temporal coordination of neuronal networks. The relative contribution of each process varies according to the affected gene, cell type, circuit, developmental stage, and functional consequence of the variant. This convergence provides the mechanistic bridge from genetic heterogeneity to the network-level pathophysiology considered in Section 4.

## 4. The Pathophysiological Core: Circuit Homeostasis, E/I Imbalance, and Network Dysregulation

The molecular mechanisms described above ultimately converge at the level of developing neural circuits. Although ion-channel dysfunction, altered synaptic transmission, receptor abnormalities, dysregulated intracellular signaling, and impaired gene-expression control originate at different molecular levels, each can interfere with the ability of neuronal networks to establish and maintain stable patterns of activity. The resulting phenotype is therefore better conceptualized as a disturbance of circuit homeostasis than as a uniform shift toward neuronal hyperexcitability. This distinction is particularly relevant to the ASD–epilepsy spectrum, in which the same genetic alteration may disturb developmental circuit homeostasis in ways that simultaneously influence seizure susceptibility, synaptic plasticity, sensory processing, cognition, and behavior [34,35].

### 4.1. Intrinsic Excitability and Failure of Network Homeostasis

Neural circuits must maintain activity within a functional dynamic range despite continuous changes in synaptic input, intrinsic membrane properties, and developmental connectivity. Homeostatic mechanisms normally compensate for such perturbations by adjusting ion-channel expression, synaptic strength, receptor composition, and neuronal firing thresholds. Genetic disruption of these processes may overwhelm or misdirect compensatory responses, producing persistent network instability. Importantly, compensatory adaptations may partially normalize average neuronal activity while introducing other abnormalities in circuit function, making it difficult to infer the network phenotype directly from the initial molecular defect [34].

Channelopathies provide a particularly clear illustration of this principle. As discussed in Section 3.1, both gain- and loss-of-function variants can destabilize neural networks, depending on the affected channel, neuronal population, subcellular compartment, and developmental stage. Importantly, the initial molecular disturbance may trigger compensatory changes in ion-channel expression, synaptic strength, and neuronal firing that partially restore average activity while altering the temporal or spatial organization of circuit function. The network phenotype therefore reflects not only the primary biophysical defect but also the capacity—and limitations—of developmental homeostatic compensation [34].

This framework also helps explain why epilepsy and neurodevelopmental impairment may coexist without being mechanistically identical. A genetic alteration that destabilizes circuit homeostasis can increase susceptibility to hypersynchronous epileptic activity while simultaneously interfering with activity-dependent processes required for normal synaptic refinement and cognitive development. Seizures may therefore represent one manifestation of network instability and may further modify developmental trajectories, rather than constituting the sole cause of neurodevelopmental dysfunction [11,34].

### 4.2. Interneuron Dysfunction and GABAergic Network Disinhibition

GABAergic interneurons are central regulators of cortical excitability, oscillatory activity, temporal precision, and experience-dependent plasticity. Despite representing a minority of cortical neurons, they comprise multiple functionally specialized populations that control distinct spatial and temporal aspects of pyramidal-cell activity. Their maturation also contributes critically to the regulation of developmental periods of enhanced cortical plasticity. Disruption of interneuron development or function can therefore have disproportionately large effects on circuit organization and information processing [35,82].

*SCN1A* provides a paradigmatic example of cell type-specific network dysfunction. Preferential impairment of NaV1.1-dependent excitability in inhibitory interneurons weakens inhibitory control and produces network disinhibition despite an underlying loss-of-function channel defect. At the circuit level, this illustrates why E/I dysregulation cannot be inferred simply from the direction of the molecular defect and why reduced excitability of one neuronal population may generate hyperexcitability of the network as a whole [34,37].

Interneuron dysfunction is not restricted to primary channelopathies. Molecular defects originating at other levels of synaptic organization can propagate through the network and secondarily alter inhibitory circuit function. *STXBP1* haploinsufficiency, for example, can compromise inhibitory neurotransmission through abnormalities involving parvalbumin- and somatostatin-expressing interneurons [59]. Experimental *SHANK3* deficiency has likewise been associated with reduced cortical interneuron activity accompanied by increased spontaneous and stimulus-evoked pyramidal-neuron firing. Selective disruption of Shank3 in cortical inhibitory interneurons can reproduce pyramidal-cell hyperactivity and sensory hyper-reactivity, illustrating how a molecular defect need not originate in a GABAergic-specific protein to produce functionally significant impairment of inhibitory circuit control [59,83].

The developmental dimension is equally important. GABAergic circuits undergo prolonged maturation and contribute to the regulation of critical periods of cortical plasticity. Alterations in interneuron maturation may therefore affect not only seizure susceptibility but also the timing of experience-dependent circuit refinement. Such disturbances provide a mechanistic link between inhibitory-circuit dysfunction, abnormal sensory processing, impaired developmental plasticity, and vulnerability to epilepsy [35,82,84].

Thus, GABAergic dysfunction in ASD–epilepsy disorders should be understood as a disturbance of when, where, and how inhibition is recruited within developing networks, rather than simply as a global reduction in inhibitory neurotransmission [34,35].

### 4.3. Synaptic Dysfunction, Network Coordination, and Impaired Plasticity

Normal neurodevelopment requires more than an appropriate average level of excitation and inhibition. Information processing depends on the precise temporal coordination of neuronal populations, appropriate synaptic strengthening and weakening, and the ability of circuits to reorganize in response to experience. Genetic abnormalities affecting synaptic proteins, neurotransmitter receptors, intracellular signaling, or transcriptional and translational regulation can disrupt these processes even when basal neuronal excitability is not markedly abnormal [34,35].

Across these molecular categories, disruption of synaptic architecture, neurotransmitter release, receptor signaling, intracellular pathways, or gene-expression programs can impair the temporal organization and adaptive modification of neuronal communication. The resulting network dysfunction may involve altered synaptic strength, mistimed recruitment of neuronal populations, impaired experience-dependent remodeling, or failure to maintain stable patterns of coordinated activity. Thus, molecularly distinct disorders can converge on common deficits in network integration and plasticity without requiring identical abnormalities of basal neuronal excitability.

These molecular disturbances can generate two apparently contrasting network consequences. On the one hand, excessive synchronization of neuronal populations can facilitate epileptiform activity and seizures. On the other, impaired physiological coordination and temporal precision may interfere with distributed information processing and developmental network integration. These phenomena are not mutually exclusive: the same network may become pathologically hypersynchronous during epileptic activity while exhibiting deficient or mistimed physiological coordination during normal cognitive and sensory processing. This distinction is particularly important in the ASD–epilepsy spectrum, because network dysfunction cannot be adequately described along a single axis of increased or decreased excitation [34,35].

For this reason, the term network desynchronization alone does not adequately describe the ASD–epilepsy phenotype. The broader concept of network dysregulation accommodates both pathological hypersynchrony associated with epilepsy and impaired physiological coordination relevant to neurodevelopmental dysfunction.

Synaptic plasticity provides a further point of convergence. Activity-dependent modification of synaptic strength is required for learning, memory, sensory adaptation, and developmental circuit refinement. Genetic disturbances that alter the timing or magnitude of plasticity may therefore produce lasting developmental effects even in the absence of continuous epileptic activity. Conversely, recurrent seizures and persistent interictal epileptiform activity may further disrupt plasticity and network maturation, particularly during sensitive developmental periods. Contemporary concepts of developmental and epileptic encephalopathy explicitly recognize these potentially separable but interacting contributions of the underlying etiology and epileptic activity [11].

Taken together, the ASD–epilepsy spectrum can be conceptualized as a disorder of developmental circuit homeostasis in which diverse molecular defects converge on abnormal regulation of neuronal excitability, inhibition, synaptic plasticity, and temporal network organization. The relative contribution of each mechanism varies according to genotype, variant function, brain region, cell type, and developmental stage. This heterogeneity helps explain why related molecular pathways may produce markedly different combinations of epilepsy, intellectual disability, language impairment, and autistic features.

At the network level, these mechanisms can be summarized as a convergent framework in which genetic alterations affecting intrinsic excitability, synaptic function, intracellular signaling, or developmental regulatory programs disrupt circuit homeostasis (Figure 1). The resulting abnormalities in E/I regulation, synaptic plasticity, and temporal coordination may diverge toward pathological hypersynchronization and epileptic activity, impaired physiological network coordination and ASD-related neurodevelopmental dysfunction, or both. Importantly, these outcomes may interact dynamically during development rather than representing a simple linear sequence from genetic defect to epilepsy and subsequently to autism.

## 5. From Gene to Bedside: Precision Therapeutics in Co-Occurring ASD and Epilepsy

The increasing identification of monogenic and pathway-defined disorders within the ASD–epilepsy spectrum is progressively shifting therapeutic strategies from predominantly syndrome-based seizure management toward mechanism-informed precision medicine. A molecular diagnosis may influence treatment at several levels: avoidance of potentially aggravating drugs, pharmacological modulation of a variant-specific functional defect, targeting of a dysregulated intracellular pathway, correction of a metabolic deficiency, or direct manipulation of gene expression or RNA processing. Precision therapy, however, should not be equated with gene-specific therapy. Rather, it encompasses a continuum of interventions that differ substantially in target specificity, level of mechanistic correction, and capacity to modify the underlying disease process.

This distinction is particularly important in developmental and epileptic encephalopathies (DEEs), in which seizure control, correction of the primary molecular abnormality, and modification of neurodevelopmental trajectory represent related but not necessarily equivalent therapeutic outcomes. Although several genetically or pathway-informed interventions have entered clinical practice and an increasing number of molecular therapies are under clinical development, the strength of evidence remains highly heterogeneous. Biological plausibility, target engagement, seizure reduction, improvement in developmental function, and true disease modification should therefore be regarded as distinct levels of therapeutic evidence.

Accordingly, the therapeutic approaches discussed below should be interpreted according to their level of clinical evidence. They range from established therapies supported by clinical trials and incorporated into current clinical practice, through mechanism-informed or off-label interventions supported primarily by observational or limited clinical evidence, to investigational therapies undergoing clinical evaluation and preclinical strategies supported predominantly by experimental data. Throughout this section, mechanistic rationale is therefore distinguished from demonstrated clinical efficacy, and early therapeutic signals are not interpreted as evidence of established disease-modifying benefit.

### 5.1. Sodium Channelopathies: Functional Variant–Guided Therapy

Voltage-gated sodium-channel disorders provide one of the clearest examples of why genetic diagnosis must be complemented by functional interpretation. Pathogenic variants in *SCN1A*, *SCN2A*, and *SCN8A* can produce markedly different—and sometimes opposing—effects on channel activity, with direct implications for treatment.

In Dravet syndrome, most pathogenic *SCN1A* variants result in loss of NaV1.1 function, with prominent impairment of inhibitory interneuron excitability. Maintenance treatment with sodium-channel-blocking antiseizure medications, particularly carbamazepine and lamotrigine, may aggravate seizures and is therefore generally avoided. Current syndrome-directed treatment includes valproate and clobazam, with stiripentol, fenfluramine, and pharmaceutical-grade cannabidiol representing additional established therapeutic options [85]. These interventions can substantially reduce seizure burden but do not directly correct the underlying *SCN1A* haploinsufficiency.

The therapeutic logic differs in *SCN2A*-related disorders. Variants associated with neonatal or early-infantile epilepsy frequently produce gain-of-function effects, and sodium-channel blockers may be particularly effective in this functional subgroup. Conversely, many loss-of-function variants are associated with later neurodevelopmental phenotypes, including intellectual disability and ASD, with or without epilepsy [39,74]. This genotype–function–phenotype relationship is clinically informative but not absolute, emphasizing that treatment decisions should integrate phenotype, developmental timing, and functional characterization when available.

A similar principle applies to *SCN8A*. Many variants associated with early-onset developmental and epileptic encephalopathy increase NaV1.6 activity, providing a mechanistic rationale for sodium-channel-blocking treatment in selected individuals. However, *SCN8A*-related disorders encompass gain-of-function, loss-of-function, and functionally complex variants associated with distinct neurological phenotypes. Recent international consensus recommendations therefore emphasize phenotype and functional variant interpretation rather than application of a uniform therapeutic strategy to all individuals with *SCN8A*-related disorders [41].

These examples illustrate a central principle of precision neurotherapeutics: identification of the affected gene defines the molecular context, but the direction, magnitude, cellular context, and developmental consequences of the functional disturbance determine how that target should be approached therapeutically. Strategies aimed at modifying sodium-channel gene expression itself are discussed in Section 5.7.

### 5.2. Potassium Channelopathies: Opportunities and Limitations of Targeted Modulation

Potassium channelopathies provide a complementary model of function-guided treatment. *KCNQ2* encodes the KV7.2 subunit contributing to the neuronal M-current. Many variants associated with severe *KCNQ2* developmental and epileptic encephalopathy reduce channel function, providing a mechanistic rationale for pharmacological enhancement of KV7 activity.

Ezogabine (retigabine), a KV7-channel opener, provided an early proof of concept. In an observational series including 11 treated individuals with *KCNQ2* encephalopathy, improvement in seizures and/or development was reported in some patients, particularly among those treated early. However, the study was retrospective and uncontrolled and therefore could not establish therapeutic efficacy [43]. Moreover, the functional heterogeneity of *KCNQ2* disorders—including gain-of-function as well as loss-of-function variants—means that channel enhancement cannot be assumed to be appropriate for every pathogenic variant.

*KCNT1* illustrates an apparently opposite therapeutic problem. Pathogenic variants associated with severe early-onset epilepsies commonly produce gain of function of the sodium-activated potassium channel KNa1.1 (Slack), providing a rationale for channel inhibition. Quinidine therefore emerged as a mechanistically attractive candidate. Although individual reports suggested seizure improvement, a small randomized, blinded, placebo-controlled crossover trial failed to demonstrate significant seizure reduction and identified dose-limiting QT prolongation [86]. Thus, *KCNT1* provides a particularly clear example in which strong molecular rationale did not translate straightforwardly into clinical efficacy.

Potassium channelopathies consequently demonstrate both the promise and limitations of precision pharmacology. Mechanistic plausibility is necessary but insufficient: variant function, pharmacokinetics and brain penetration, developmental timing, off-target effects, and clinical safety all determine whether a molecularly rational intervention translates into meaningful clinical benefit.

### 5.3. GABAergic Dysfunction and Receptor-Targeted Therapies

Disorders affecting GABAergic neurotransmission appear intuitively suited to enhancement of inhibitory signaling, but the mechanistic framework developed in Section 3 and Section 4 indicates substantially greater complexity. *GABRB3*-associated disorders, for example, encompass heterogeneous functional abnormalities involving receptor expression, trafficking, gating, GABA sensitivity, and current kinetics. A gene-level diagnosis therefore does not establish a uniform pharmacological direction.

Enhancement of GABA_A-receptor activity may be mechanistically appropriate in selected contexts, but it cannot be assumed to correct all disorders involving GABAergic signaling. Functional characterization and knowledge of the affected receptor population, neuronal context, and developmental stage are therefore important both for genotype–phenotype interpretation and for the rational design of receptor-targeted interventions.

Ganaxolone provides a clinically established example of a related but less gene-specific strategy. Ganaxolone is a neuroactive steroid positive allosteric modulator of GABA_A receptors. In the randomized, placebo-controlled phase 3 Marigold trial, ganaxolone significantly reduced major motor seizure frequency in patients with CDKL5 deficiency disorder [87]. This represents syndrome- and pathway-informed treatment rather than direct correction of the causal *CDKL5* defect and illustrates an important distinction between precision treatment of downstream network dysfunction and molecular correction of the primary etiology. The underlying *CDKL5*-related developmental biology is discussed in Section 3.2 [60,61].

### 5.4. Targeting Intracellular Signaling: mTOR Pathway Modulation

The PI3K–AKT–mTOR pathway provides one of the strongest examples of successful pathway-directed treatment in genetic neurodevelopmental disease. Loss of *TSC1* or *TSC2* function results in excessive mTORC1 activity, creating a direct molecular rationale for mTOR inhibition [62,63].

Everolimus has demonstrated antiseizure efficacy in TSC-associated treatment-resistant focal-onset seizures. In the randomized, double-blind, placebo-controlled phase 3 EXIST-3 trial, adjunctive everolimus significantly reduced seizure frequency compared with placebo, providing high-level evidence that pharmacological targeting of a genetically dysregulated intracellular pathway can translate into clinically meaningful seizure reduction [88].

However, correction of pathway activity sufficient to improve seizure control should not automatically be equated with normalization of neurodevelopment. Studies of neuropsychiatric and cognitive outcomes with mTOR inhibition have yielded more limited and heterogeneous findings. Even in TSC—one of the clearest examples of pathway-directed precision treatment—target engagement, seizure reduction, and developmental benefit therefore remain distinct therapeutic outcomes [63,89].

TSC also illustrates the importance of therapeutic timing through a different mechanism. In the EPISTOP study, infants with TSC underwent serial EEG surveillance, and preventive vigabatrin initiated after detection of epileptiform EEG activity but before clinical seizures delayed seizure onset and reduced the risks of clinical seizures, drug-resistant epilepsy, and infantile spasms compared with conventional treatment [90]. EPISTOP does not demonstrate complete prevention of neurodevelopmental impairment, nor is vigabatrin a direct mTOR-correcting therapy. Rather, it provides evidence that intervening against emerging epileptic network dysfunction before recurrent clinical seizures may modify the epilepsy trajectory.

### 5.5. Glutamatergic and Synaptic Targets

NMDAR-related disorders provide a particularly instructive example of variant-specific pharmacological reasoning. Pathogenic variants in *GRIN* genes can produce gain- or loss-of-function effects, and treatment strategies should therefore ideally be informed by functional characterization rather than gene identity alone [50].

Memantine, an NMDAR antagonist, initially provided proof of concept in individual patients with functionally characterized gain-of-function *GRIN* variants. More recent retrospective evidence has extended this principle: in a 2026 cohort of 34 individuals with *GRIN* variants, clinical benefit from memantine was substantially more frequent among individuals with functionally classified gain-of-function variants than among those with loss-of-function, indeterminate, or functionally neutral variants, although responses remained heterogeneous [91,92]. These findings strengthen the principle of function-guided therapy while remaining insufficient to establish memantine as universally effective treatment for *GRIN*-related disorders.

Conversely, L-serine supplementation has been investigated as a strategy for GRIN loss-of-function disorders, based on enhancement of NMDAR co-agonist availability. Initial individualized observations in *GRIN2B* loss-of-function disease suggested improvement in motor, cognitive, and communication outcomes [93]. Subsequent open-label studies, including a phase 2A study of children with *GRIN* loss-of-function variants, have provided additional evidence of tolerability and signals of improvement in selected functional outcomes, while emphasizing the need for controlled validation [93,94].

Synaptic disorders have also stimulated attempts to modulate downstream signaling rather than the primary genetic defect. In Phelan–McDermid syndrome, recombinant human insulin-like growth factor-1 (IGF-1) was evaluated in a small pilot controlled trial, with preliminary improvement in selected social and repetitive-behavior measures [95]. Subsequent studies have explored growth hormone as an indirect strategy for increasing IGF-1 signaling. A 2022 proof-of-concept study by Sethuram et al. reported feasibility and exploratory clinical signals, but these approaches remain investigational and the small sample sizes preclude definitive conclusions [96].

These studies illustrate both the potential and limitations of synaptic precision therapy: molecularly rational interventions can generate clinically relevant signals, but small cohorts, functional heterogeneity, variable outcome measures, and limited controlled evidence frequently prevent firm conclusions about efficacy or disease modification.

### 5.6. Metabolic and Cofactor-Responsive Disorders

Metabolic disorders provide some of the clearest examples in which an etiological diagnosis can directly alter treatment. This section uses cerebral creatine deficiency syndromes as an illustrative model rather than as an exhaustive review of metabolic causes of epilepsy and neurodevelopmental dysfunction. Numerous other treatable inherited metabolic disorders can present with developmental impairment, epilepsy, and behavioral or autistic features, but their systematic discussion is beyond the scope of this genetically focused review [97]. Cerebral creatine deficiency syndromes comprise arginine:glycine amidinotransferase deficiency (GATM/AGAT), guanidinoacetate methyltransferase deficiency (GAMT), and creatine transporter deficiency (*SLC6A8*). These disorders may present with intellectual disability, severe speech impairment, autistic features, movement abnormalities, and epilepsy, yet their therapeutic responsiveness differs substantially according to the affected metabolic step [98].

In GAMT deficiency, creatine supplementation combined with strategies to reduce guanidinoacetate accumulation can substantially improve clinical outcome, particularly when treatment begins early. AGAT deficiency is likewise responsive to creatine replacement and represents an especially compelling example of the potential value of presymptomatic or very early etiological diagnosis [98,99].

In contrast, *SLC6A8*-related creatine transporter deficiency is considerably more difficult to treat because the primary defect involves cellular creatine transport, including transport into the brain. Creatine supplementation alone or in combination with precursor amino acids has produced variable and generally incomplete clinical responses, emphasizing that biochemical knowledge of a deficient pathway does not necessarily translate into effective substrate replacement when transport itself is impaired [100].

These disorders demonstrate particularly clearly why early etiological diagnosis matters. In selected metabolic diseases, treatment before substantial developmental dysfunction has accumulated may fundamentally alter outcome, whereas later biochemical correction may improve specific functions without fully reversing established neurodevelopmental abnormalities [98,99,100].

### 5.7. Gene- and Molecularly Targeted Therapies

The most direct form of precision therapy aims to modify expression of the disease-causing gene or its transcript. Antisense oligonucleotides (ASOs), RNA-based strategies, gene replacement, gene activation, and related molecular approaches are rapidly expanding the therapeutic landscape of genetic neurodevelopmental disorders. Unlike conventional antiseizure treatments, these strategies have the potential to intervene upstream of network dysfunction and therefore raise the possibility of modifying both epilepsy and associated developmental manifestations [89].

Dravet syndrome now provides an important example of an investigational molecularly targeted therapy. Zorevunersen is an investigational ASO designed to increase productive *SCN1A* expression from the unaffected allele and thereby increase NaV1.1 expression. In 2026, Laux et al. reported phase 1–2a open-label studies in children and adolescents with Dravet syndrome, with safety and pharmacokinetics as principal objectives and exploratory evidence of reductions in convulsive seizure frequency and improvement in measures of adaptive behavior. These early clinical findings support continued investigation of zorevunersen as a potential disease-modifying therapy but do not establish definitive efficacy, long-term clinical benefit, or an established disease-modifying treatment [101].

A parallel investigational molecular strategy has entered early-phase clinical evaluation in Angelman syndrome. In neurons, the paternal *UBE3A* allele is normally transcriptionally silenced by the *UBE3A* antisense transcript (*UBE3A*-ATS).

Rugonersen is an investigational ASO designed to bind and reduce *UBE3A*-ATS, thereby releasing this antisense-mediated silencing and increasing expression of the otherwise inactive paternal *UBE3A* allele. In the phase 1, open-label TANGELO trial, rugonersen was administered to children with Angelman syndrome and showed an acceptable safety and tolerability profile, dose-dependent partial normalization of the characteristic EEG abnormality, and exploratory signals of clinical improvement beyond expectations from natural-history data [102]. These findings support continued clinical development and demonstrate the feasibility of molecular reactivation of paternal *UBE3A* in humans; however, controlled evidence is still required to establish clinical efficacy, durability of benefit, and whether the intervention provides disease-modifying effects.

Gene- and RNA-targeted therapies should consequently be regarded as a heterogeneous and rapidly evolving therapeutic class, ranging from preclinical proof-of-concept strategies to clinically advanced investigational programs with early human efficacy signals, rather than as established disease-modifying therapies for genetic ASD–epilepsy disorders [89].

An additional challenge for gene- and RNA-targeted therapies is effective delivery to the central nervous system (CNS). Systemically administered molecular therapies may be limited by blood–brain barrier (BBB) penetration, peripheral exposure, and off-target effects, whereas direct CNS delivery can improve target exposure but introduces procedural and distributional constraints. These challenges are particularly relevant to nucleic acid-based therapies, including antisense oligonucleotides, for which systemic CNS delivery remains difficult because of both the BBB and the physicochemical properties of oligonucleotides [103]. Intrathecal or intracerebroventricular administration can bypass the BBB and facilitate CNS exposure, but therapeutic efficacy still depends on adequate biodistribution and uptake within disease-relevant brain regions and cell populations [103]. Similar considerations apply to CNS-directed gene therapy, in which route of administration, vector tropism, tissue and cell-type specificity, peripheral exposure, and distribution within the CNS influence both efficacy and safety [104]. Thus, successful molecular correction depends not only on target specificity but also on achieving sufficient and appropriately distributed CNS exposure, while the optimal route, dose, durability, and timing of intervention may differ according to the therapeutic platform and developmental stage.

### 5.8. The Clinical Correlation Paradox: Why Seizure Control Does Not Necessarily Normalize Neurodevelopment

The development of precision therapies exposes a fundamental clinical question in genetic ASD–epilepsy disorders: if seizures and neurodevelopmental impairment arise from a shared molecular defect, why does successful seizure control not consistently produce parallel improvement in cognition, language, or autistic features?

The concept of developmental and epileptic encephalopathy provides part of the answer. Neurodevelopmental impairment may reflect both direct developmental consequences of the underlying etiology and additional adverse effects of epileptic activity. Their relative contributions differ among disorders, individuals, and developmental stages. Seizure suppression may therefore have markedly different neurodevelopmental consequences depending on when, where, and to what extent epileptic activity participates in the disease process [11].

A second component of the paradox is developmental timing. Correction of a primary molecular defect after abnormal circuit maturation has occurred may improve neuronal function without fully reversing changes established during earlier sensitive periods. Dose, tissue distribution, cell-type specificity, degree of molecular correction, and especially the developmental stage at which treatment is delivered may therefore influence whether gene- or RNA-targeted therapy produces seizure reduction, broader neurodevelopmental benefit, or both [105,106].

This review proposes three complementary mechanistic paradigms to conceptualize this heterogeneity. These paradigms are intended as a conceptual framework rather than as established diagnostic categories.

#### 5.8.1. Electrical Interference Paradigm

In the Electrical Interference Paradigm, epileptic seizures or persistent interictal epileptiform activity contribute directly to dysfunction of a developing network that retains substantial developmental potential. Abnormal electrical activity during sensitive periods may interfere with language acquisition, learning, memory, synaptic plasticity, and experience-dependent circuit refinement. Under these circumstances, early and effective suppression of pathological activity may permit meaningful developmental improvement [11,106].

The epilepsy–aphasia spectrum associated with *GRIN2A* and developmental and/or epileptic encephalopathy with spike-wave activation in sleep (DEE-SWAS) provide representative examples. In these disorders, the timing, distribution, and burden of epileptiform activity may contribute substantially to cognitive and language dysfunction, making rapid suppression of pathological electrical activity an important potential component of developmental rescue [10,51,52].

TSC is a particularly informative, but not pure, example of the Electrical Interference Paradigm. A systematic review and meta-analysis found that ASD in children with TSC was associated with seizures and, more specifically, with infantile spasms compared with other seizure types [107]. Longitudinal evidence published in 2026 further identified developmental trajectories in which infantile spasms, alone and in combination with greater cortical tuber burden, were associated with later autistic features in children with TSC [108]. These findings support the possibility that early severe epileptic activity may contribute to, or amplify, neurodevelopmental risk. However, they do not establish a direct causal relationship between infantile spasms and autism in TSC. In a prospective infant cohort, epilepsy was associated with increased ASD risk, but atypical developmental trajectories were already detectable before seizure onset in a subgroup, and early versus conventional vigabatrin treatment did not reduce ASD risk at 24 months [109]. EPISTOP nevertheless demonstrated that vigabatrin initiated after emergence of epileptiform EEG abnormalities but before clinical seizures reduced subsequent epilepsy-related outcomes, including infantile spasms [90]. Thus, early suppression of epileptic activity may potentially modify one component of neurodevelopmental risk, while primary mTOR-related developmental abnormalities remain independently relevant.

#### 5.8.2. Kinetic Race Paradigm

In the Kinetic Race Paradigm, therapeutic outcome depends on the temporal competition between progressive pathological neurodevelopment and effective intervention. The primary molecular defect initiates abnormal cellular and circuit development, but there may be a developmental window during which correction can prevent or substantially reduce the accumulation of persistent dysfunction.

Treatable metabolic encephalopathies provide particularly clear examples. In GAMT and AGAT deficiencies, early metabolic correction can markedly improve outcome, whereas treatment initiated after prolonged developmental impairment may correct or improve the biochemical disturbance without fully reversing established cognitive, language, or behavioral abnormalities [98,99].

TSC may also partially overlap with this paradigm because epileptiform abnormalities and seizures frequently emerge during infancy, when neural circuits are undergoing rapid maturation. Earlier intervention may therefore have greater potential to preserve developmental function than later suppression of established epilepsy, although the extent to which seizure prevention or early seizure control independently modifies neurodevelopmental outcome remains uncertain. The critical variable is not simply whether an intervention is mechanistically appropriate, but when it is delivered relative to the evolving developmental pathology [64,90].

The same principle is increasingly relevant to molecular therapies. The early clinical experience with zorevunersen and rugonersen demonstrates that primary molecular abnormalities can now be manipulated in humans, but whether earlier correction will yield greater developmental rescue remains a central unanswered question [101,102].

#### 5.8.3. Independent Dual-Track Paradigm

In the Independent Dual-Track Paradigm, epilepsy and ASD-related neurodevelopmental dysfunction are partially parallel consequences of the same underlying genetic alteration. The molecular defect affects multiple cellular or circuit processes, some of which promote seizures while others independently disrupt cognition, communication, behavior, or developmental plasticity.

This model may be particularly relevant to disorders involving synaptic and transcriptional regulators such as *SHANK3*, *SYNGAP1*, *MECP2*, *UBE3A*, and *FMR1*. As discussed in Section 3, these disorders can directly perturb synaptic maturation, activity-dependent plasticity, transcriptional or translational regulation, and circuit development. Seizures may therefore represent one consequence of the underlying molecular disorder rather than the necessary mediator of the developmental phenotype. Effective seizure control can consequently reduce one component of disease burden without correcting developmental abnormalities produced directly by the primary genetic defect [55,56,68,69,71].

Clinical observations from the previously reported pediatric ASD cohort provide additional support for this heterogeneous relationship between seizure control and neurodevelopmental outcome. Among children with ASD and epilepsy, 58% achieved seizure freedom and a further 27% experienced a >50% reduction in seizure frequency; however, improved seizure control was not accompanied by a corresponding improvement in autistic behavior [7]. These observations cannot establish mechanistic independence between epilepsy and ASD, but they illustrate that successful suppression of seizures does not necessarily translate into improvement in autistic behavior. This clinical dissociation is consistent with the possibility that, in at least a subset of individuals, epilepsy and ASD represent partially parallel manifestations of shared neurodevelopmental pathology.

#### 5.8.4. Integrating the Three Paradigms

These three paradigms should not be regarded as mutually exclusive diagnostic categories. Rather, they represent complementary positions within a mechanistic continuum, and a single genetic disorder—or even an individual patient—may incorporate more than one mechanism at different developmental stages.

TSC provides a particularly informative example. Primary TSC1/TSC2–mTOR dysregulation directly alters brain development, cellular growth, and synaptic function, while structural abnormalities and emerging epileptic activity provide additional sources of network dysfunction. TSC may therefore combine features of the Independent Dual-Track, Electrical Interference, and Kinetic Race paradigms, with their relative contributions changing across development [62,64,90].

More broadly, the therapeutic implications of these paradigms differ. When epileptic activity is a major developmental disruptor, rapid seizure and EEG control may itself provide substantial developmental benefit. When the underlying molecular defect progressively alters circuit maturation, treatment may need to be delivered within an early developmental window. When epilepsy and neurodevelopmental dysfunction arise through partially parallel consequences of the same genetic defect, seizure suppression alone is unlikely to normalize cognition or autistic features, and upstream molecular or pathway-directed therapy may be required.

The central objective of precision therapeutics in the ASD–epilepsy spectrum should therefore extend beyond seizure reduction. A mechanistically informed strategy requires identification of which component of the disease process is being targeted, at what developmental stage, and with which expected outcome. Genetic diagnosis, functional variant interpretation, biomarkers of target engagement and network dysfunction, and developmentally sensitive clinical endpoints will increasingly need to be integrated if emerging molecular therapies are to achieve meaningful modification of both epileptic and neurodevelopmental trajectories.

The principal features and therapeutic implications of these three mechanistic paradigms are summarized in Table 4.

This distinction has major implications for precision therapeutics. Treatment success should not be defined solely by seizure freedom. For molecular and gene-targeted interventions, clinically meaningful outcomes should also include cognition, language, adaptive behavior, social communication, motor development, and quality of life, assessed over sufficiently long periods to detect changes in developmental trajectory [105,106]. Conversely, failure to normalize neurodevelopment despite excellent seizure control should not automatically be interpreted as failure of antiseizure treatment; it may instead indicate that the neurodevelopmental phenotype is driven substantially by the underlying molecular disorder [10,106].

Ultimately, the relationship between epilepsy and ASD is better conceptualized as a dynamic developmental interaction between shared genetic vulnerability, intrinsic network dysfunction, epileptic activity, therapeutic timing, and developmental plasticity, rather than as a simple unidirectional causal sequence. This framework reconciles shared molecular mechanisms with the variable clinical relationship between seizure burden and autistic or cognitive outcomes and provides a rationale for therapeutic strategies that address both epileptic activity and the underlying neurodevelopmental disorder.

## 6. Limitations of the Review

This review has several limitations inherent to its narrative design. Study identification and selection were relevance-based rather than systematic, and no formal risk-of-bias assessment or quantitative evidence synthesis was performed. Consequently, the selection and relative weighting of individual studies may have been influenced by author judgment, and the evidence discussed should not be interpreted as an exhaustive or systematically graded representation of the literature. In addition, the substantial heterogeneity of genetic disorders, phenotypic definitions, study designs, and therapeutic endpoints across the ASD–epilepsy spectrum limits direct comparison between studies.

## 7. Conclusions and Future Directions

The co-occurrence of ASD and epilepsy is increasingly understood as a complex neurodevelopmental spectrum arising from partially shared genetic and biological mechanisms. Advances in genomic medicine have revealed convergence across apparently distinct molecular disorders, including ion-channel dysfunction, abnormalities of synaptic organization and neurotransmission, altered receptor signaling, dysregulation of intracellular pathways, and disruption of transcriptional and translational control. At the systems level, these mechanisms converge on impaired circuit homeostasis, altered excitation–inhibition regulation, disrupted synaptic plasticity, and abnormal network dynamics. This convergence provides a mechanistic framework for understanding why epilepsy, ASD-related features, intellectual disability, and other neurodevelopmental manifestations frequently coexist, while remaining highly variable across individuals and genetic disorders [5,6].

Genetic diagnosis is therefore becoming an increasingly important component of clinical characterization in individuals with co-occurring epilepsy and ASD, particularly when accompanied by early seizure onset, intellectual disability, developmental regression, dysmorphism, or other neurological abnormalities. However, identification of a pathogenic variant should be regarded as the beginning rather than the endpoint of precision medicine. Interpretation increasingly requires consideration of variant-specific functional effects, cell-type and circuit vulnerability, developmental stage, and the extent to which the identified molecular mechanism is therapeutically accessible. Genomic information can therefore refine and complement clinical syndromic classification rather than simply replace it [6,13,14,15,16].

The therapeutic consequences of this molecular stratification are already evident. Function-guided treatment of ion channelopathies, pathway-directed therapy in mTOR-related disorders, correction of selected metabolic defects, and emerging gene- and RNA-targeted approaches demonstrate that molecular diagnosis can influence therapeutic decision-making. Nevertheless, the evidence supporting these strategies remains heterogeneous. For many proposed precision therapies, clinical experience is still based on small cohorts, observational studies, or early-phase trials, and mechanistic plausibility should not be equated with demonstrated clinical efficacy or disease modification [89].

A major challenge for the next generation of precision therapeutics will therefore be to determine whether correction of a molecular defect can alter neurodevelopmental trajectory rather than merely reduce seizures or modify an electrophysiological or molecular biomarker. This distinction is particularly important in DEEs, in which developmental impairment may reflect both the direct effects of the underlying etiology and the additional consequences of epileptic activity [10,106]. The therapeutic window may differ substantially among disorders: in some conditions, early suppression of epileptic activity may preserve developmental function, whereas in others, intervention may need to precede overt seizures or substantial circuit maturation to achieve meaningful disease modification.

The development of reliable biomarkers represents a parallel priority. EEG measures, quantitative neuroimaging, molecular biomarkers, digital phenotyping, and other physiological readouts may provide intermediate indicators of target engagement and network response. However, improvement in a biomarker should not automatically be interpreted as evidence of meaningful neurodevelopmental benefit. Biomarkers will be most valuable when validated against longitudinal cognitive, behavioral, language, motor, and adaptive outcomes and when their relationship to clinically meaningful change is established [105,106].

Clinical-trial design must evolve in parallel with these therapeutic advances. Conventional seizure-frequency endpoints remain essential but are insufficient for interventions intended to modify the underlying neurodevelopmental disorder. Trials should incorporate developmentally appropriate measures of cognition, communication, adaptive behavior, motor function, behavior, and quality of life, together with sufficiently long follow-up to detect changes in developmental trajectory. Robust natural-history data and controls, age-dependent endpoints, and strategies to address floor effects in individuals with profound impairment will be particularly important. Recent work on operational definitions of DEEs similarly emphasizes the need for precisely defined populations and targeted outcome measures as precision therapies enter clinical trials [106].

Finally, the rapid expansion of molecular therapeutics raises important practical challenges. Access to genomic diagnosis, functional testing, multidisciplinary expertise, and advanced therapies remains uneven, while increasingly individualized treatments may be associated with substantial financial and logistical demands. The future of precision medicine in ASD–epilepsy disorders will therefore depend not only on therapeutic innovation but also on international collaboration, standardized outcome assessment, long-term safety surveillance, and equitable access to diagnosis and treatment [89,105].

In conclusion, genetic convergence between ASD and epilepsy does not imply a single molecular mechanism or a uniform therapeutic strategy. Rather, diverse genetic alterations converge on common disturbances of developing neural circuits, while the resulting clinical phenotype is shaped by variant function, cell type, developmental timing, network plasticity, and epileptic activity. The central challenge for precision therapeutics is therefore no longer simply to identify which gene is affected, but to determine how, where, and when the molecular defect alters the developing brain—and when that process remains therapeutically modifiable. Addressing these questions will be essential if precision medicine is to progress from seizure-directed treatment toward genuine modification of neurodevelopmental outcome.

## Figures and Tables

**Figure 1:**
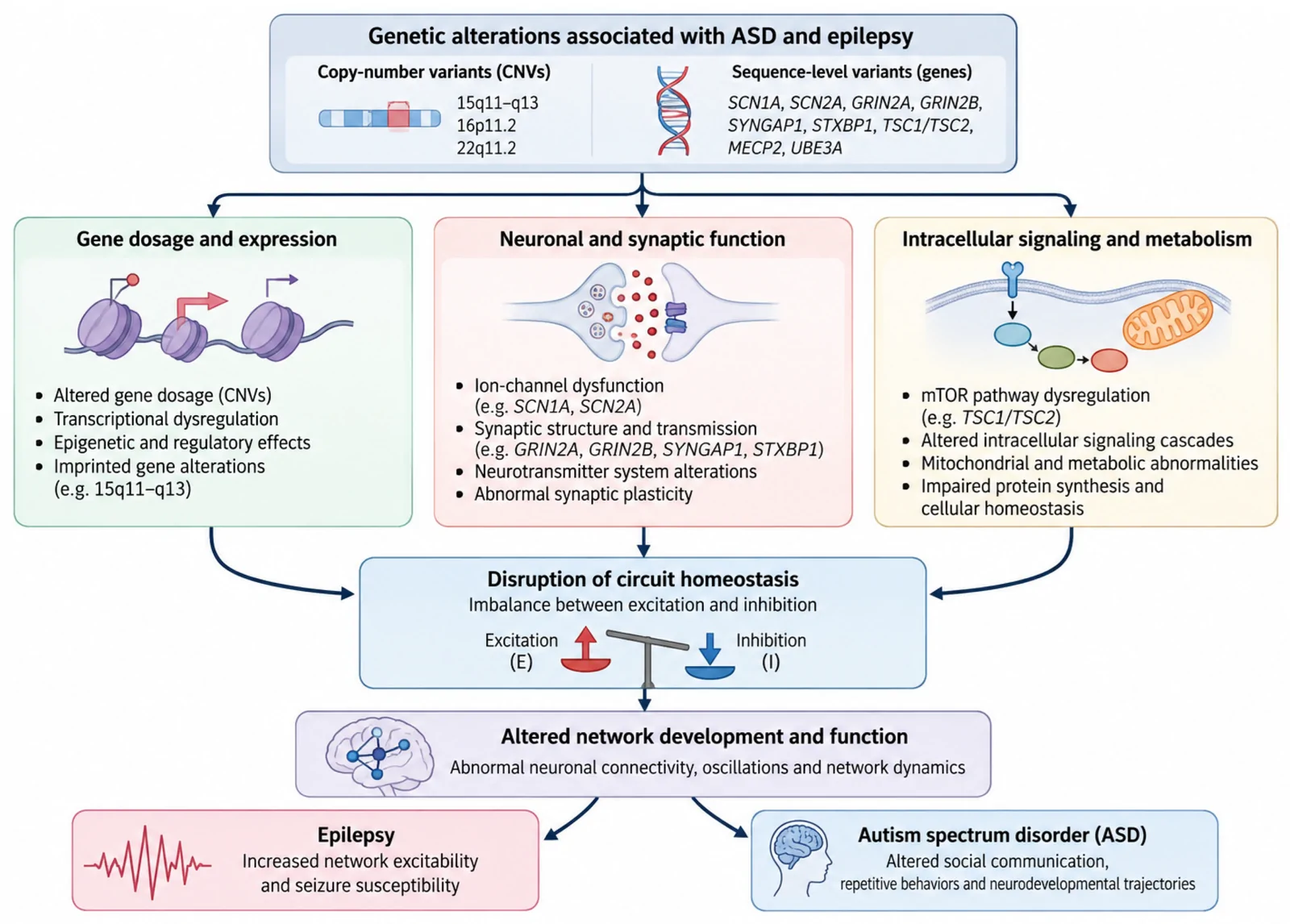
Convergent molecular, cellular, and network mechanisms linking genetic alterations to epilepsy and ASD-related neurodevelopmental dysfunction. Pathogenic genetic alterations, including sequence variants, copy-number variants (CNVs), and regulatory defects, may affect three interconnected mechanistic domains: intrinsic neuronal excitability, illustrated by ion-channel genes (*SCN1A*, *SCN2A*, *SCN8A*, *KCNQ2*, and *KCNT1*); synaptic dysfunction and plasticity, involving synaptic proteins, neurotransmitter receptors, and release mechanisms (*SHANK3*, *SYNGAP1*, *STXBP1*, *GRIN2A*, *GRIN2B*, and *GABRB3*); and developmental/regulatory pathways involving intracellular signaling and transcriptional or translational regulation (*TSC1/TSC2*, *PTEN*, *CHD8*, *MECP2*, *UBE3A*, and *FMR1*). These mechanisms converge on disrupted circuit homeostasis, including abnormal excitation–inhibition (E/I) regulation and impaired synaptic plasticity and temporal coordination, resulting in abnormal network dynamics. Network dysfunction may diverge toward pathological hypersynchrony and epileptic discharges/seizures, impaired physiological network coordination and ASD-related neurodevelopmental dysfunction, or both. Bidirectional arrows between epileptic activity and ASD-related neurodevelopmental dysfunction indicate potential mutual developmental modulation rather than a uniform causal relationship, as epileptic activity may further affect neurodevelopmental networks while underlying neurodevelopmental dysfunction may also modify seizure susceptibility and network organization. Developmental stage, genetic background, seizures, experience, inflammation, and other environmental or biological modifiers may influence both phenotypic trajectories. Arrows indicate proposed functional relationships and mechanistic convergence rather than a strictly linear causal sequence. ASD, autism spectrum disorder; CNV, copy-number variant; E/I, excitation–inhibition.

**Table 1:** Shared and distinctive clinical manifestations relevant to the ASD–epilepsy spectrum.

Clinical Domain	ASD	Epilepsy/Genetically Defined DEEs	Interpretation in the ASD–Epilepsy Spectrum
**Core manifestations**	Persistent deficits in social communication and social interaction together with restricted/repetitive patterns of behavior, interests, or activities [1]	Epilepsy is diagnosed according to established clinical criteria reflecting an enduring predisposition to generate epileptic seizures [8]	The core diagnostic manifestations are distinct; epilepsy is a comorbidity rather than a defining feature of ASD [1,8]
**Intellectual/developmental impairment**	Intellectual disability may co-occur with ASD but is not required for the diagnosis [1]	Intellectual and developmental impairment is prominent in many genetically defined DEEs [10]	Intellectual disability is one of the most consistently demonstrated modifiers of epilepsy risk in ASD [2,3]
**Developmental regression**	Developmental regression occurs in a subset of individuals with ASD and most commonly involves loss of previously acquired language and/or social skills [9]	Developmental slowing or regression may occur in DEEs [10,11]	In genetic DEEs, the underlying molecular defect may itself contribute to developmental impairment; developmental abnormalities should therefore not automatically be attributed to epileptic activity alone [11]
**Additional neurological manifestations**	Core ASD diagnostic criteria concern social-communication/social-interaction deficits and restricted/repetitive patterns of behavior rather than neurological motor manifestations [1]	Motor abnormalities, hypotonia, movement disorders, and other neurological manifestations may occur within the broader phenotype of genetically defined DEEs [10]	When present with autistic features and epilepsy, additional neurological manifestations should be interpreted in the context of the broader neurological/genetic phenotype [10,12]

Abbreviations: ASD, autism spectrum disorder; DEE, developmental and epileptic encephalopathy.

**Table 2:** Common recurrent copy-number variants associated with ASD, epilepsy, and related neurodevelopmental phenotypes.

CNV Region	Type	Major Dosage-Sensitive/Candidate Genes	ASD/Autistic Phenotype	Epilepsy/Seizure Phenotype	Other Major Neurodevelopmental Features	References
**1q21.1**	Deletion	Multigenic region; *PRKAB2*, *CHD1L*, and *BCL9* among candidate genes	ASD/autistic features occur in a subset; incomplete penetrance	Seizures occur in a subset of carriers	DD/ID, learning difficulties, microcephaly, motor impairment, and hypotonia; marked variable expressivity	[20]
**1q21.1**	Duplication	Multigenic region; *PRKAB2*, *CHD1L*, and *BCL9* among candidate genes	Increased frequency of ASD and greater ASD symptom severity have been reported compared with deletion carriers	Seizures have been reported, although the epilepsy phenotype is less well characterized	DD/ID, learning difficulties, macrocephaly, motor impairment, and hypotonia; variable expressivity	[20]
**15q11–q13**	Maternal duplication/ idic(15)	*UBE3A*; GABA-receptor gene cluster including *GABRB3*, *GABRA5*, and *GABRG3*	Strong association with ASD, particularly for maternally derived duplications	Epilepsy is common, particularly in idic(15); multiple seizure types and infantile spasms may occur	Hypotonia, DD/ID, and marked speech/language impairment	[17,27]
**15q13.3**	Deletion	*CHRNA7*, *OTUD7A*	ASD occurs in a subset; incomplete penetrance	Strong association with epilepsy/seizures; generalized epilepsy is an important component of the phenotypic spectrum	DD/ID, speech/language impairment, and variable behavioral and psychiatric manifestations	[21]
**16p11.2 BP4–BP5**	Deletion	Multigenic region; *PRRT2*, *KCTD13*, *TAOK2*, and *MAPK3* among candidate genes	Well-established association with ASD and broader neurodevelopmental phenotypes	Seizures and epilepsy occur in a substantial subset; epilepsy phenotypes are heterogeneous	Speech/language impairment, DD/ID, hypotonia and motor abnormalities, macrocephaly, and increased obesity risk	[18,24,25]
**16p11.2 BP4–BP5**	Duplication	Same multigenic dosage-sensitive interval	Increased risk of ASD and other neurodevelopmental disorders, with variable penetrance	Seizures and epilepsy occur in a subset; frequency and neurological phenotype differ from those observed in deletion carriers	Speech/language impairment, DD/ID, motor abnormalities, microcephaly, and psychiatric manifestations	[18,24,25]
**16p13.11**	Deletion	*NDE1* and other genes within the recurrent interval	ASD and broader neurodevelopmental phenotypes have been reported	Established recurrent susceptibility CNV for genetic generalized epilepsies	DD/ID, speech/language impairment, and variable neuropsychiatric manifestations	[22,28]
**22q11.2**	Deletion	Multigenic region; *TBX1*, *DGCR8*, *COMT*, and *PRODH* among relevant genes	ASD/autistic phenotypes occur in a subset	Increased seizure susceptibility; acute symptomatic seizures, particularly those associated with hypocalcemia, should be distinguished from epilepsy	Cognitive/developmental impairment, ADHD and other psychiatric manifestations, and multisystem congenital abnormalities	[19,29]
**22q11.2**	Duplication	Reciprocal multigenic 22q11.2 interval	Increased rate of ASD, with marked variable expressivity and incomplete penetrance	Seizures and epilepsy have been reported, but available evidence is limited and epilepsy does not appear to be a consistent feature of the duplication syndrome	DD/ID, speech/language impairment, hypotonia, and variable behavioral, psychiatric, and congenital manifestations; mildly affected or unaffected carriers may occur	[26,30]
**22q13.3**	Deletion (Phelan–McDermid syndrome)	*SHANK3* is a major contributor; additional genes within larger deletions may modify the phenotype	ASD/autistic features are prominent, although formal assessment may be complicated by severe developmental impairment	Epilepsy occurs in a substantial subset; seizure semiology and severity are heterogeneous	Global DD/ID, absent or severely impaired speech, hypotonia, and motor impairment	[23,31]

Abbreviations: ASD, autism spectrum disorder; CNV, copy-number variant; DD, developmental delay; ID, intellectual disability; idic(15), isodicentric chromosome 15.

**Table 3:** Molecular and cellular mechanisms of representative genes converging on the ASD–epilepsy spectrum.

Gene/Representative OMIM Phenotype (MIM no.)	Molecular Pathway/Principal Function	Variant-Associated Mechanism	ASD–Epilepsy Mechanistic Convergence	Representative Clinical Phenotype
***SCN1A***—Dravet syndrome (#607208)	NaV1.1 voltage-gated Na+ channel; action-potential generation, particularly in inhibitory interneurons	Predominantly LoF/haploinsufficiency in Dravet syndrome; functional effects vary across *SCN1A* disorders	Reduced excitability of inhibitory interneurons disrupts inhibitory control and E/I regulation, promoting seizures and potentially affecting developmental circuit function	Epilepsy is predominant; Dravet syndrome commonly includes ID/developmental impairment and may include autistic features/ASD [37,38]
***SCN2A***—Developmental and epileptic encephalopathy 11 (#613721)	NaV1.2 voltage-gated Na+ channel; excitability of developing and mature excitatory neurons	Early-onset epilepsy commonly associated with GoF; LoF/truncating variants strongly associated with ASD/ID	Opposite functional effects within the same channel alter neuronal excitability and developmental circuit maturation, providing a direct genotype–function–phenotype relationship	Strong convergence: early GoF variants predominantly cause epilepsy/DEE, whereas LoF variants are predominantly associated with ASD/ID, often without early epilepsy [39,40]
***SCN8A***—Developmental and epileptic encephalopathy 13 (#614558); cognitive impairment with or without cerebellar ataxia (#614306)	NaV1.6 voltage-gated Na+ channel; action-potential initiation and propagation, particularly at axon initial segments and nodes	GoF common in *SCN8A*-DEE; LoF/reduced function associated with some NDD phenotypes	Altered intrinsic excitability disrupts network stability and developmental circuit function; consequences depend on functional direction and neuronal context	Epilepsy/DEE is prominent in GoF disorders; ID, autistic features/ASD, or movement abnormalities may occur with or without epilepsy [41,42]
***KCNQ2***—Developmental and epileptic encephalopathy 7 (#613720)	KV7.2 K+ channel; neuronal M-current, membrane-potential stabilization, and limitation of repetitive firing	LoF/dominant-negative variants common in DEE; GoF variants also occur	Abnormal M-current alters neuronal excitability and activity-dependent maturation during early network development	Spectrum ranges from self-limited neonatal epilepsy to severe DEE with DD/ID and variable autistic features [43,44,45,46]
***KCNT1***—Developmental and epileptic encephalopathy 14 (#614959)	KNa1.1/Slack Na+-activated K+ channel; regulation of firing adaptation, repolarization, and activity-dependent signaling	Predominantly GoF in severe early-onset epilepsies	Abnormal firing dynamics and activity-dependent signaling destabilize network activity and may also interfere with developmental circuit function	Severe early-onset epilepsy/DEE predominates; profound developmental impairment is common, whereas ASD-specific involvement is less consistently defined [47,48,49]
***GRIN2A***—Focal epilepsy with speech disorder, with or without impaired intellectual development (#245570)	GluN2A NMDAR subunit; excitatory transmission, synaptic maturation, and activity-dependent plasticity	Both GoF and LoF	Altered NMDAR signaling affects synaptic plasticity, language-network maturation, and neuronal excitability	Predominantly epilepsy–aphasia/speech-language phenotype; ASD/autistic features are less consistently expressed [50,51,52]
***GRIN2B***—Intellectual developmental disorder, autosomal dominant 6, with or without seizures (#613970); developmental and epileptic encephalopathy 27 (#616139)	GluN2B NMDAR subunit; early neuronal differentiation, synaptogenesis, and synaptic plasticity	Both GoF and LoF	Abnormal NMDAR signaling disrupts early neuronal/synaptic development and plasticity while variably altering network excitability	Broad NDD with DD/ID and behavioral/ASD-related features; epilepsy is variably present [50,53]
***GABRB3***—Developmental and epileptic encephalopathy 43 (#617113)	GABA\(_A\) receptor β3 subunit; inhibitory neurotransmission and developmental circuit regulation	Functionally heterogeneous, including GoF and LoF effects and altered gating, trafficking, expression, or GABA sensitivity	Either direction of receptor dysfunction may disturb developmental E/I regulation, demonstrating that the phenotype cannot be reduced to deficient inhibition alone	Spectrum from epilepsy to severe DEE with ID and behavioral/autistic features; phenotype varies according to functional effect [54]
***SHANK3***—Phelan–McDermid syndrome (#606232)	Postsynaptic scaffold; organization of excitatory postsynaptic density, glutamatergic transmission, and plasticity	Haploinsufficiency/LoF	Disrupted postsynaptic organization, receptor function, and plasticity impair developmental circuit organization; epileptic instability occurs only in a subset	ASD/autistic phenotype and severe speech/developmental impairment are prominent; epilepsy is variable [55]
***SYNGAP1***—Intellectual developmental disorder, autosomal dominant 5 (#612621)	Postsynaptic Ras/Rap GAP; regulation of excitatory-synapse maturation and critical-period plasticity	Haploinsufficiency/LoF	Premature excitatory-synapse maturation and impaired critical-period plasticity jointly disturb cognition/behavior and network excitability	Strong NDD–epilepsy convergence: ID/DD and epilepsy are common, with frequent autistic features [56,57,58]
***STXBP1***—Developmental and epileptic encephalopathy 4 (#612164)	Munc18-1; presynaptic vesicle fusion and neurotransmitter release	Predominantly haploinsufficiency/LoF	Impaired neurotransmitter release, including vulnerability of inhibitory circuitry, disturbs E/I regulation and developmental network function	Epilepsy/DEE and ID/DD are prominent; behavioral/autistic features occur, but a specific ASD mechanism is less clearly defined [59]
***CDKL5***—Developmental and epileptic encephalopathy 2 (#300672)	Serine/threonine kinase; neuronal maturation, dendritic morphogenesis, synapse formation, and synaptic function	Predominantly LoF/reduced kinase activity	Primary disruption of neuronal and synaptic maturation coexists with profound epileptic network dysfunction; developmental impairment is not explained by seizures alone	Very-early-onset severe DEE with profound NDD; autistic features may occur, although formal ASD assessment may be difficult [60,61]
***TSC1/TSC2***—Tuberous sclerosis 1 (#191100)/tuberous sclerosis 2 (#613254)	Hamartin–tuberin complex; negative regulation of mTORC1 signaling	LoF → mTORC1 hyperactivation	Excess mTOR signaling alters neuronal growth, migration, synaptic development, plasticity, and excitability, providing shared substrates for ASD/NDD and epileptogenesis	TSC frequently includes epilepsy, ID, and ASD/TAND; relative contributions of primary mTOR dysregulation and epileptic activity vary [62,63,64]
***PTEN***—Macrocephaly–autism syndrome (#605309)	PTEN phosphatase; negative regulation of PI3K–AKT–mTOR signaling	LoF → pathway hyperactivation	Excess growth signaling alters neuronal size, migration, connectivity, synaptic plasticity, and network excitability	Macrocephaly and ASD/NDD are prominent in neurodevelopmental presentations; seizures/epilepsy occur in a subset [65,66,67]
***MECP2***—Rett syndrome (#312750)	Methyl-CpG-binding protein 2; activity-dependent transcriptional regulation and neuronal maturation	Predominantly LoF in Rett syndrome	Broad transcriptional dysregulation alters synaptic maturation, plasticity, and E/I regulation, affecting both developmental function and network stability	Rett syndrome with severe NDD and frequent epilepsy; autistic features may occur, particularly during specific developmental stages [68]
***UBE3A***—Angelman syndrome (#105830)	E3 ubiquitin ligase; protein homeostasis and activity-dependent synaptic regulation	Loss of maternal neuronal UBE3A in Angelman syndrome; dosage-sensitive effects	Abnormal protein homeostasis and synaptic plasticity disturb circuit maturation and excitability	Angelman syndrome includes severe DD/ID and frequent epilepsy; autistic features may occur, while increased UBE3A dosage is associated with ASD-related phenotypes [69,70]
***FMR1***—Fragile X syndrome (#300624)	FMRP RNA-binding protein; activity-dependent translational regulation	Loss of FMRP, usually through *FMR1* silencing	Dysregulated local protein synthesis alters synaptic maturation, plasticity, and network homeostasis	Fragile X syndrome commonly includes ID and ASD/autistic features; epilepsy occurs in a subset [71,72]
***CHD8***—Intellectual developmental disorder with autism and macrocephaly (#615032)	ATP-dependent chromatin remodeler; transcriptional regulation of neurodevelopmental programs	Haploinsufficiency/LoF	Broad transcriptional dysregulation affects neuronal differentiation, synaptic development, and circuit function; direct convergence with epileptogenesis is weaker than for many other genes listed	Strong ASD/NDD association, often with macrocephaly and language/developmental impairment; epilepsy is not a defining feature [73]

Abbreviations: ASD, autism spectrum disorder; DD, developmental delay; DEE, developmental and epileptic encephalopathy; E/I, excitation–inhibition; GoF, gain of function; ID, intellectual disability; LoF, loss of function; NDD, neurodevelopmental disorder; NMDAR, N-methyl-D-aspartate receptor; TAND, tuberous sclerosis-associated neuropsychiatric disorders.

**Table 4:** Proposed conceptual mechanistic framework for the variable relationship between seizure control and neurodevelopmental outcome in genetic ASD–epilepsy disorders.

Dimension	Electrical Interference Paradigm	Kinetic Race Paradigm	Independent Dual-Track Paradigm
Core concept	Epileptic and/or interictal epileptiform activity directly interferes with ongoing developmental network function	Outcome depends on the timing of effective etiological or mechanism-directed treatment relative to progressive developmental circuit dysfunction	Epilepsy and ASD-related neurodevelopmental dysfunction arise partly as parallel consequences of the same molecular defect
Role of the primary genetic defect	Establishes susceptibility to abnormal network activity; developmental impairment may be substantially amplified by epileptic activity [10,11]	Initiates pathological development whose consequences become progressively less reversible with time [82,84,98,99]	Directly affects multiple developmental pathways, only some of which are responsible for epilepsy [10,11,55,56,64,68,69,71]
Role of epileptic activity	Major potentially modifiable contributor to cognitive, language, or behavioral dysfunction [10,11,90]	Important contributor whose impact depends strongly on developmental timing [82,84,90,98,99]	Variable or secondary; seizure burden may be only partially related to core neurodevelopmental impairment [7,10,11]
Expected effect of seizure control	Potentially substantial developmental benefit when epileptic activity is suppressed early and effectively [10,11,90]	Greatest benefit when intervention precedes increasingly fixed network changes; diminishing developmental rescue with delay [82,84,98,99]	Seizure improvement may occur without proportional improvement in ASD-related or cognitive manifestations [7,10,11]
Importance of therapeutic timing	High, particularly during sensitive developmental periods [82,84]	Central determinant of outcome [82,84,98,99]	Relevant, but seizure timing alone may not determine developmental outcome [10,11]
Representative examples	*GRIN2A*-related epilepsy–aphasia spectrum; DEE-SWAS; TSC with infantile spasms as a potentially modifiable developmental amplifier [10,51,52,90,107,108,109]	GATM (AGAT) and GAMT deficiencies; TSC may show overlapping features [90,98,99]	*SHANK3*, *SYNGAP1*, *MECP2*, *UBE3A*, and *FMR1* disorders; TSC may also exhibit dual-track features [55,56,64,68,69,71]
Precision-therapy implication	Rapid suppression of pathological electrical activity may preserve developmental function [10,11,90]	Early etiological diagnosis and intervention may be required for meaningful disease modification [98,99]	Therapy may need to target the primary molecular/developmental defect in addition to controlling seizures [10,11,89]
Key therapeutic endpoint	Seizure/EEG improvement plus developmental recovery or stabilization [10,11,106]	Preservation or redirection of developmental trajectory [98,99,106]	Independent assessment of seizure and neurodevelopmental outcomes [10,11,105,106]

Abbreviations: ASD, autism spectrum disorder; DEE, developmental and epileptic encephalopathy; DEE-SWAS, developmental and/or epileptic encephalopathy with spike-wave activation in sleep; EEG, electroencephalography; TSC, tuberous sclerosis complex. Note: The three paradigms represent conceptual and potentially overlapping mechanistic models proposed to facilitate interpretation of clinical and experimental observations; they are not validated clinical or diagnostic classifications. Evidence designations indicate the nature of the supporting evidence for each representative example and do not constitute a formal evidence-grading system.

## Data Availability

No new data were created or analyzed in this study. Data sharing is not applicable to this article.

## Abbreviations

The following abbreviations are used in this manuscript:

**Abbreviation**	**Definition**
AGAT	Arginine:glycine amidinotransferase
ASD	Autism spectrum disorder
ASO	Antisense oligonucleotide
CDD	CDKL5 deficiency disorder
CMA	Chromosomal microarray analysis
DEE	Developmental and epileptic encephalopathy
DEE-SWAS	Developmental and/or epileptic encephalopathy with spike-wave activation in sleep
EEG	Electroencephalography
E/I	Excitation/inhibition
FMRP	Fragile X messenger ribonucleoprotein
GABA	Gamma-aminobutyric acid
GAMT	Guanidinoacetate methyltransferase
GoF	Gain of function
ID	Intellectual disability
IGF-1	Insulin-like growth factor-1
LoF	Loss of function
mTOR	Mechanistic target of rapamycin
mTORC1	Mechanistic target of rapamycin complex 1
NDD	Neurodevelopmental disorder
NGS	Next-generation sequencing
NMDAR	N-methyl-D-aspartate receptor
PI3K	Phosphoinositide 3-kinase
RNA	Ribonucleic acid
TSC	Tuberous sclerosis complex
UBE3A-ATS	UBE3A antisense transcript
WES	Whole-exome sequencing
WGS	Whole-genome sequencing
